# Atypical antipsychotics in bipolar disorder: systematic review of randomised trials

**DOI:** 10.1186/1471-244X-7-40

**Published:** 2007-08-16

**Authors:** Sheena Derry, R Andrew Moore

**Affiliations:** 1 Pain Research and Nuffield Department of Anaesthetics, University of Oxford, Oxford Radcliffe Hospitals, The Churchill, Headington, Oxford, OX3 7LJ, UK

## Abstract

**Background:**

Atypical antipsychotics are increasingly used for treatment of mental illnesses like schizophrenia and bipolar disorder, and considered to have fewer extrapyramidal effects than older antipsychotics.

**Methods:**

We examined efficacy in randomised trials of bipolar disorder where the presenting episode was either depression, or manic/mixed, comparing atypical antipsychotic with placebo or active comparator, examined withdrawals for any cause, or due to lack of efficacy or adverse events, and combined all phases for adverse event analysis. Studies were found through systematic search (PubMed, EMBASE, Cochrane Library), and data combined for analysis where there was clinical homogeneity, with especial reference to trial duration.

**Results:**

In five trials (2,206 patients) participants presented with a depressive episode, and in 25 trials (6,174 patients) the presenting episode was manic or mixed.

In 8-week studies presenting with depression, quetiapine and olanzapine produced significantly better rates of response and symptomatic remission than placebo, with NNTs of 5–6, but more adverse event withdrawals (NNH 12). With mania or mixed presentation atypical antipsychotics produced significantly better rates of response and symptomatic remission than placebo, with NNTs of about 5 up to six weeks, and 4 at 6–12 weeks, but more adverse event withdrawals (NNH of about 22) in studies of 6–12 weeks. In comparisons with established treatments, atypical antipsychotics had similar efficacy, but significantly fewer adverse event withdrawals (NNT to prevent one withdrawal about 10). In maintenance trials atypical antipsychotics had significantly fewer relapses to depression or mania than placebo or active comparator.

In placebo-controlled trials, atypical antipsychotics were associated with higher rates of weight gain of ≥7% (mainly olanzapine trials), somnolence, and extrapyramidal symptoms. In active controlled trials, atypical antipsychotics were associated with lower rates of extrapyramidal symptoms, but higher rates of weight gain and somnolence.

**Conclusion:**

Atypical antipsychotics are effective in treating both phases of bipolar disorder compared with placebo, and as effective as established drug therapies. Atypical antipsychotics produce fewer extrapyramidal symptoms, but weight gain is more common (with olanzapine). There is insufficient data confidently to distinguish between different atypical antipsychotics.

## Background

Bipolar disorder is now recognised as a potentially treatable psychiatric illness with substantial morbidity and mortality and high social and economic impact [1]. There is no cure, and every aspect of its definition, mechanisms and treatment is subject to debate. Moreover, bipolar disorder is common, with an estimated lifetime prevalence of 2%in a recent Canadian study [2], and of 3% for bipolar I disorder in a US study [3].

Therapies must address the control of acute episodes (manic, depressed or mixed), and maintenance of remission of symptoms. Drug treatments have included lithium, anticonvulsants, and antipsychotics, but current therapies have proven inadequate for many patients; only half of bipolar patients achieve remission over two years, and half of these relapse within the two years [4]. Issues in drug treatment involve not only efficacy, but also tolerability. Adverse events, including extrapyramidal symptoms and weight gain, can be significant and influence adherence.

Newer (atypical) antipsychotics are generally considered to have fewer extrapyramidal effects. They have proven efficacy in treatment of acute mania and schizophrenia [5] and have also been used in dementia [6]. Newer drugs are often subjected to more, better, and more detailed investigation in randomised trials than older medicines.

Given the likely nature of randomised trials available, the aim of this review was to:

1. Examine the efficacy in randomised trials of atypical antipsychotics where the presenting episode is depression or manic/mixed, comparing atypical antipsychotic with placebo or active comparator.

2. Examine withdrawals for any cause, or due to lack of efficacy or adverse events.

3. Combine all phases for adverse event analysis.

Potential sources of clinical heterogeneity in the studies are types of patient, severity and duration of symptoms, drug and dose used, the duration of therapy and/or study, and the aim of therapy, whether for treatment of acute symptoms or maintenance of remission. In addition there may be differences in which outcomes were measured and reported.

## Methods

We searched PubMed, EMBASE and the Cochrane Library up to December 2006 for randomised controlled trials using atypical antipsychotic drugs to treat bipolar disorder. The search strategy used individual drug names, "bipolar" and "random*", together with appropriate indexing terms for bipolar disorder and randomised controlled trial.

For inclusion trials had to be randomised and double blind, and use an atypical antipsychotic drug alone or in combination with a mood stabilising drug such as lithium, valproate, divalproex, lamotrigine, or carbamazepine to treat adult patients with documented bipolar disorder, with either a placebo or active comparator. Trials had to have a minimum of 10 patients per treatment arm, and a planned duration of at least three weeks. The abstracts were read, and potentially useful reports retrieved in full paper copy. Decisions on inclusion or exclusion were made by consensus. No information was taken from posters or abstracts, and studies were read carefully to avoid including duplicate material. Studies were scored for reporting quality using a common method [7] utilising reporting of randomisation, blinding and withdrawals. The maximum score possible was 5 points, and no study could be included with fewer than 2 points (one for randomisation and one for blinding).

Information extracted from the trials included details of the patients (number, age, sex, nature of presenting episode), treatment regimens and concomitant medications. We used the number of patients randomised and receiving at least a single dose of drug in order to have an intention to treat analysis; almost all outcomes were reported in this way. Outcomes of efficacy, tolerability and harm, and switching to the opposite state/pole were extracted, using dichotomous data wherever possible. For efficacy we particularly sought information on response and/or remission, and for harm, information on weight gain, extrapyramidal symptoms, and changes in prolactin, glucose and lipid levels.

Guidelines for quality of reporting of meta-analyses were followed where appropriate [8]. The prior intention was to pool data where there was clinical and methodological homogeneity, with similar patients, dose, duration, outcomes, and comparators, but not where numbers of events were small, and random chance could dominate effects of treatment [9]. Homogeneity tests and funnel plots, though commonly used in meta-analysis, were not used here because they have been found to be unreliable [10-12]. Instead clinical homogeneity was examined graphically [13]. Relative benefit (or risk) and number-needed-to-treat or harm (NNT or NNH) were calculated with 95% confidence intervals. Relative benefit or risk was calculated using a fixed effects model [14], with no statistically significant difference between treatments assumed when the 95% confidence intervals included unity. We added 0.5 to treatment and comparator arms of trials in which at least one arm had no events. Number-needed-to-treat (or harm) was calculated by the method of Cook and Sackett [15] using the pooled number of observations only when there was a statistically significant difference of relative benefit or risk (where the confidence interval did not include 1). Statistical significance of any difference between numbers needed to treat for different drugs was assumed if there was no overlap of the confidence intervals, and additionally tested using the z statistic [16].

The following terms were used to describe adverse outcomes in terms of harm or prevention of harm [17]:

• When significantly fewer adverse events occurred with atypical antipsychotic than with control (placebo or active) we used the term the number-needed-to-treat to prevent one event (NNTp).

• When significantly more adverse events occurred with atypical antipsychotic compared with control (placebo or active) we used the term the number-needed-to-harm to cause one event (NNH).

We chose only to pool data for analysis if there were at least two trials and at least 250 patients [9]. We chose to analyse according to comparator (placebo or active) and trial duration, separating short-term trials of less than six weeks, from those of six to 12 weeks. Longer duration trials involved maintenance therapy following response to treatment, and these were also analysed separately as trials of longer than 12 weeks.

## Results

We found five trials [18-22] in which the participants presented with a depressive episode, and 25 [23-49] (two reported separately at two time points) in which the presenting episode was manic or mixed. All had industry sponsorship. Details of the included studies together with outcome data extracted from the studies are provided for presenting episode of depression [see Additional file 1] and mania/mixed [see Additional file 2], as well as individual adverse events [see Additional file 3], and a list of excluded studies [see Additional file 4].

Reported outcomes were measured using some kind of scale (depression or mania rating scales, weight, cholesterol levels) [see Additional files 1 and 2], while other outcomes, predominantly treatment emergent adverse events, were elicited from patients as subjective evaluations [see Additional file 3]. A few outcomes were reported both using scale measurements and subjective evaluations. Wherever the distinction was clear, both sets of data are presented.

## Efficacy

### Presenting episode: depression

Five trials reported on 1,739 patients, 2206 of whom were treated with an atypical antipsychotic. Mean ages in the trials were 36 to 42 years, and just under half (44%) of patients were men. Patients were diagnosed as Bipolar I [18,21] or Bipolar I or II [19,20,22], and in one trial [20] patients were excluded if they had failed to respond to at least two classes of antidepressant in the current episode. The trials were of mixed reporting quality, with one scoring 5, and three 4, and one 3, out of a maximum 5 points.

Most patients (80%) were in three large placebo-controlled trials [18,20,23] lasting eight weeks, one comparing olanzapine monotherapy or olanzapine plus fluoxetine with placebo, and the others comparing quetiapine monotherapy, at different target dosages, with placebo. One, small (30 patients), placebo-controlled trial lasting 12 weeks [19], examined a mood stabiliser together with risperidone, paroxetine or a combination of the two. The remaining patients were in an active controlled trial comparing olanzapine plus fluoxetine with lamotrigine [21]. All trials permitted limited use of benzodiazepines for the first three to four weeks of treatment. The numbers of patients treated with each drug are in Table 1, and dosage, mean daily doses of trial drugs elsewhere [see Additional file 1].

**Table 1:** Numbers of patients treated with different drugs in trials of atypical antipsychotics in bipolar disorder

**Index episode and duration**	**Drug**	**Number of Patients**
Depression		
8–12 weeks	olanzapine	351
	olanzapine/fluoxetine	287
	quetiapine	661
	risperidone + ms	10
	risperidone/paroxetine + ms	10
	paroxetine + ms	10
	lamotrigine	205
	placebo	685

Mania/Mixed		
<6 weeks	olanzapine	265
	risperidone	449
	risperidone + ms	127
	quetiapine	209
	quetiapine + ms	275
	ziprasidone	270
	aripiprazole	437
	divalproex	126
	haloperidol	428
	haloperidol + ms	53
	lithium	128
	placebo	1126
	placebo + ms	411

6–12 weeks	olanzapine	291
	olanzapine + ms	229
	quetiapine	209
	aripiprazole	174
	divalproex	63
	lithium	98
	haloperidol	488
	placebo	196
	placebo + ms	115

>12 weeks	olanzapine	567
	olanzapine + ms	51
	aripipazole	78
	divalproex	126
	lithium	214
	placebo	219
	placebo + ms	48

ms: mood stabiliser (lithium, valproate, divalproex, carbamazepine)

Response to treatment was generally defined as ≥50% reduction in depression rating scale measurement, remission as ≤12 on MADRS or ≤7 on HAM-D, and emergence of/switch to mania as YMRS ≥15 or 16. Results for the three trials with placebo-only control groups [18,20,22] are in Table 2 with analysis for individual monotherapy using titrated doses of olanzapine [18] and quetiapine monotherapy [20,22] against placebo combined and separately, but omitting olanzapine plus fluoxetine [18] where there were fewer than 100 patients treated. For both response and remission, all treatments were significantly better than placebo, with a number-needed-to-treat (NNT) of about 4–5 for quetiapine, and about 12 for olanzapine alone; quetiapine was significantly better than olanzapine. The combined NNT was about 6 for response and 5 for remission. The rate of switch into a manic state was low (2–6%), and not significantly different from placebo (4–7%) for either treatment.

**Table 2:** Outcomes for placeo controlled trials in bipolar depression [18, 20, 22] – efficacy and discontinuations in trials lasting 8 weeks

		**Events/total patients**		**Event rate (%)**			
				
**Outcome**	**Trials**	**Treatment**	**Placebo**	**Treatment**	**Placebo**	**Relative Risk (95% CI)**	**NNT (95% CI)**
**Efficacy**							
							
**Response**							
olanzapine 2–20 mg/day	1	137/351	108/355	39	30	1.3 (1.04 to 1.6)	11.6 (6.4 to 62)
quetiapine 300–600 mg/day	2	379/648	313/330	58	40	1.5 (1.3 to 1.7)	5.4 (4.2 to 7.7)
Combined atypical	3	516/999	240/685	52	35	1.4 (1.2 to 1.6)	6.0 (4.8 to 7.7)
**Symptomatic remission**							
olanzapine 2–20 mg/day	1	115/351	87/355	33	25	1.3 (1.06 to 1.7)	12.1 (6.7 to 62)
quetiapine 300–600 mg/day	2	640/648	96/330	52	29	1.8 (1.5 to 2.2)	4.3 (3.5 to 5.9)
Combined atypical	3	455/999	183/685	46	27	1.6 (1.3 to 1.9)	5.3 (4.3 to 7.0)
**Emergence of mania**							
olanzapine 2–20 mg/day	1	19/345	23/345	6	7	0.8 (0.5 to 1.5)	not calculated
quetiapine 300–600 mg/day	2	23/697	18/347	5	3	0.6 (0.4 to 1.2)	not calculated
Combined atypical	3	42/1042	41/692	4	6	0.7 (0.5 to 1.1)	not calculated

**Discontinuations**							
							
**All cause**							
olanzapine 2–20 mg/day	1	191/351	232/355	54	65	0.8 (0.7 to 0.9)	**9.1 (5.5 to 27)**
quetiapine 300–600 mg/day	2	292/702	132/349	38	42	1.1 (0.8 to 1.3)	not calculated
Combined atypical	3	483/1053	364/704	46	52	0.9 (0.8 to 1.05)	**17 (10 to 50)**
**Lack of efficacy**							
olanzapine 2–20 mg/day	1	73/351	121/355	21	34	0.6 (0.5 to 0.8)	**7.5 (5.1 to 15)**
quetiapine 300–600 mg/day	2	13/702	37/349	2	11	0.2 (0.1 to 0.3)	**11 (7.7 to 20)**
Combined atypical	3	86/1053	158/704	8	22	0.5 (0.4 to 0.6)	**7.0 (5.6 to 10)**
**Adverse events**							
olanzapine 2–20 mg/day	1	34/351	19/355	10	5	1.8 (1.1 to 3.1)	23 (12 to 220)
quetiapine 300–600 mg/day	2	109/702	17/349	16	5	3.2 (2.0 to 5.2)	9.4 (6.7 to 14)
Combined atypical	3	143/1053	36/704	14	5	2.6 (1.8 to 3.7)	12 (10 to 14)

For discontinuations NNTp is shown in **bold**, indicating fewer events with treatment than placebo. More events with treatment than placebo, NNH, is in plain text. The trial with olanzapine included only the monotherapy treatment arm

All cause discontinuations were significantly less common for olanzapine than placebo. Discontinuations for lack of efficacy were significantly less common in all active treatment groups than placebo, with NNTps of about 7 to 11; the combined NNT to prevent one lack of efficacy discontinuation was 7 (95% confidence interval 5 to 9). Discontinuations for adverse events were significantly more common for olanzapine alone and quetiapine than for placebo (NNH 23 for olanzapine, 9 for quetiapine).

There were two trials with an active control. One [19] had only ten patients in each treatment group, and the other [21] demonstrated no large difference between lamotrigine and olanzapine plus fluoxetine.

### Presenting episode: mania or mixed

Twenty-five trials [23-49] reported on a total of 6,174 patients, 3,226 of whom were treated with an atypical antipsychotic. Mean ages in trials were generally 35 to 43 years, and about half of patients were men (33 to 62% in individual trials). Six trials specifically excluded patients who had a history of intolerance to the experimental, or similar, drugs [27,30,33,45,46,48] six excluded those with a history of poor response [28,34,37,38,40,44], and six excluded those with a history of either intolerance or poor response [31,32,42,43,47,49]. In addition, five trials excluded patients with rapid cycling [31,36,37,43,44]. Sixteen trials only reported outcomes at less than six weeks (mostly three weeks). Six trials [39-44] reported at six to 12 weeks (three reporting results also at three weeks in the same report). Five trials [45-49] reported at times longer than 12 weeks, (26 to 78 weeks); two of these trials had previously reported three week results in separate papers included in the 13 papers of less than six week results. Papers were of good to high reporting quality, with one scoring 2, 13 scoring 3, nine 4, and four 5, out of a maximum 5 points. Points were lost due to inadequate descriptions of randomisation/allocation or blinding methods; all described withdrawals and dropouts.

Trials compared atypical antipsychotic as monotherapy or in combination with a mood stabiliser, with placebo, mood stabiliser monotherapy, or other active treatment (divalproex or haloperidol). All trials permitted limited use of benzodiazepines, usually with tapering dose over the first two weeks, and all but four [30,32,44,49] permitted use of anticholinergics for treatment of extrapyramidal symptoms; prophylactic use was not permitted in any trial. Details of dosage, mean daily doses of trial drugs, and concomitant medication are in Additional file 2 [see Additional file 2].

Table 1 shows the number of patients treated with each drug, for periods of less than six weeks, 6–12 weeks, and for longer than 12 weeks. The figures in Table 1 are larger than the total number of patients because some trials reported outcomes after more than one time. Because some trials were only placebo-controlled, and others only active-controlled, there were limits on the amount of information available for analysis.

#### Response

**Figure 1:**
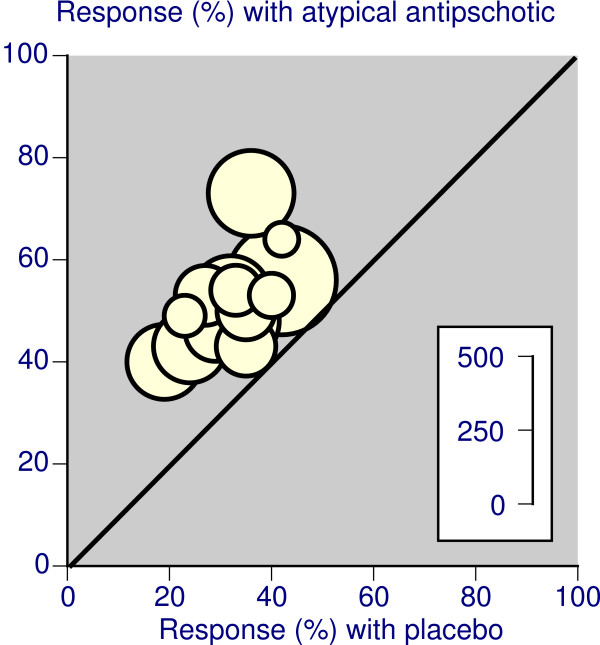
Response rates with atypical antipsychotic and placebo in placebo controlled trials lasting less than six weeks, where the presenting episode was mania or mixed. The inset scale relates the number of patients in the comparison.

**Figure 2:**
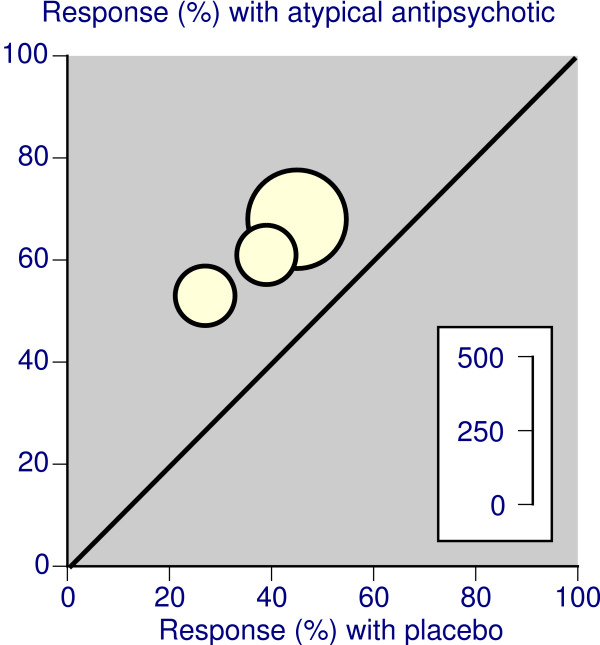
Response rates with atypical antipsychotic and placebo in placebo controlled trials lasting 6–12 weeks, where the presenting episode was mania or mixed. The inset scale relates the number of patients in the comparison.

Response to treatment was generally described as ≥50% decrease in YMRS score (or equivalent) from baseline. For placebo controlled trials lasting less than six weeks, there was remarkable consistency for response between different treatment regimens (Figure 1). Overall, for over 3,000 patients treated with either atypical antipsychotic or placebo, the relative risk was 1.6 (95%CI 1.5 to 1.8), with an NNT of 5.1 (4.4 to 6.2). Results for individual drugs and combined therapy with a mood stabiliser had NNTs between 4.3 and 6.1 (Table 3). In placebo controlled trials lasting 6 to 12 weeks involving over 700 patients (Table 3), the relative risk was 1.6 (1.4 to 1.9) and the NNT 4.0 (3.1 to 5.6), again with very similar results in individual trials (Figure 2).

**Table 3:** Outcomes for placebo controlled trials in bipolar mania (<6 weeks and 6–12 weeks)

		**Events/total patients**		**Event rate (%)**			
				
**Outcome**	**Trials**	**Treatment**	**Placebo**	**Treatment**	**Placebo**	**Relative Risk (95% CI)**	**NNT (95% CI)**
**Less than 6 weeks [24, 26, 28, 29, 31-38, 42, 43]**							
**Efficacy**							
							
**Response**							
All mono- and adjunctive therapy	14	845/1634	473/1467	52	32	1.6 (1.5 to 1.8)	5.1 (4.4 to 6.2)
All monotherapy only	8	486/949	234/791	51	30	1.7 (1.5 to 2.0)	4.6 (3.8 to 5.8)
Olanzapine	2	69/125	41/129	55	32	1.8 (1.3 to 2.4)	4.3 (2.8 to 8.7)
Risperidone	4	276/502	157/478	55	33	1.7 (1.5 to 2.0)	4.5 (3.6 to 6.2)
Quetiapine	4	247/474	167/469	52	36	1.5 (1.3 to 1.7)	6.1 (4.4 to 9.8)
Aripiprazole	2	123/263	66/259	47	25	1.8 (1.4 to 2.3)	4.7 (3.4 to 7.6)
**Symptomatic remission**							
All mono- and adjunctive therapy	4	210/448	128/453	47	28	1.7 (1.4 to 2.0)	5.4 (4.0 to 8.1)
All monotherapy only	2	83/182	44/179	46	25	1.9 (1.4 to 2.5)	4.8 (3.3 to 8.8)
**Emergence of depression**							
Quetiapine, plus mood stabiliser	2	33/275	30/285	12	11	1.2 (0.7 to 1.8)	not calculated

**Discontinuations**							
							
All cause	13	513/1507	613/1350	34	45	0.7 (0.7 to 0.8)	**8.8 (6.7 to 13)**
Lack of efficacy	13	187/1507	306/1350	12	23	0.5 (0.5 to 0.6)	**9.8 (7.7 to 13)**
Adverse events	13	81/1507	66/1350	5.4	4.9	1.1 (0.8 to 1.5)	not calculated

**6 to 12 weeks [39, 42,43]**							
**Efficacy**							
							
**Response**							
All mono- and adjunctive therapy	3	268/428	116/309	63	38	1.6 (1.4 to 1.9)	4.0 (3.1 to 5.6)
**Symptomatic remission**							
All mono- and adjunctive therapy	3	309/428	145/309	72	47	1.5 (1.3 to 1.7)	4.0 (3.1 to 5.5)
**Emergence of depression**							
All mono- and adjunctive therapy	2	9/208	17/197	8.6	4.3	0.5 (0.2 to 1.1)	not calculated

**Discontinuations**							
							
All cause	3	151/438	154/313	34	49	0.8 (0.6 to 0.9)	**6.8 (4.6 to 13)**
Lack of efficacy	3	50/438	95/313	11	30	0.5 (0.3 to 0.6)	**5.3 (4.0 to 7.7)**
Adverse events	3	37/438	12/313	8.4	3.8	2.2 (1.1 to 4.4)	22 (13 to 80)

For discontinuations NNTp is shown in **bold**, indicating fewer events with treatment than placebo. More events with treatment than placebo, NNH, is in plain text

**Figure 3:**
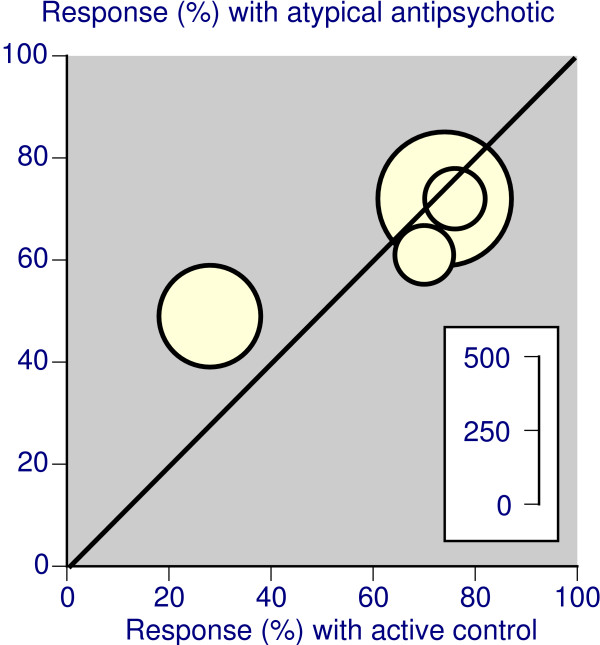
Response rates with atypical antipsychotic and comparator in active controlled trials lasting 6–12 weeks, where the presenting episode was mania or mixed. The inset scale relates the number of patients in the comparison.

For active controlled trials there were data for over 900 patients in trials lasting less than six weeks (Table 4), and over 1,200 patients in trials lasting 6 to 12 weeks (Table 4), with no significant difference between treatments. One study [44] individually showed aripipazole to be better than haloperidol, but response rates in that trial were low (Figure 3).

**Table 4:** Outcomes for active controlled trials in bipolar mania (<6 weeks and 6–12 weeks)

		**Events/total patients**		**Event rate (%)**			
				
**Outcome**	**Trials**	**Treatment**	**Active control**	**Treatment**	**Active control**	**Relative Risk (95% CI)**	**NNT (95% CI)**
**Less than 6 weeks [37, 42, 43]**							
**Efficacy**							
							
All mono- and adjunctive therapy	3	242/487	227/466	50	49	1.0 (0.9 to 1.2)	not calculated
**Symptomatic remission**							
All mono- and adjunctive therapy	3	137/333	126/322	41	39	1.1 (0.9 to 1.3)	not calculated

**Discontinuations**							
							
All cause	4	75/346	90/338	22	27	0.8 (0.6 to 1.1)	not calculated
Lack of efficacy	3	19/331	17/323	5.7	5.3	1.1 (0.6 to 2.1)	not calculated
Adverse events	4	21/346	15/338	6.1	4.4	1.4 (0.7 to 2.6)	not calculated

**6 to 12 weeks [40-44]**							
**Efficacy**							
							
All mono- and adjunctive therapy	4	394/616	353/585	64	60	1.1 (0.97 to 1.2)	not calculated
**Symptomatic remission**							
All mono- and adjunctive therapy	4	335/616	280/585	54	48	1.1 (1.01 to 1.3)	15 (8.2 to 110)
**Emergence of depression**							
All mono- and adjunctive therapy	4	40/510	62/492	7.8	13	0.6 (0.4 to 0.9)	21 (12 to 99)

**Discontinuations**							
							
All cause	5	300/675	346/651	44	53	0.8 (0.8 to 0.9)	**11 (7.1 to 30)**
Lack of efficacy	5	119/675	94/651	18	14	1.2 (0.95 to 1.6)	not calculated
Adverse events	5	68/675	132/651	10	20	0.5 (0.4 to 0.7)	**9.8 (7.1 to 16)**

For discontinuations NNTp is shown in **bold**, indicating fewer events with treatment than placebo. More events with treatment than placebo, NNH, is in plain text. Active comparators were haloperidol or lithium

Only two trials reported on time to response. In one [27] median response time was significantly shorter for olanzapine than divalproex, and in the other [39] it was shorter for olanzapine plus mood stabiliser than for placebo plus mood stabiliser (18 vs 28 days).

Trials lasting longer than 12 weeks enrolled patients who had already responded to treatment, and so response was not an outcome measured or reported in these trials.

#### Remission

Remission was generally described as YMRS score of ≤12. In placebo controlled trials lasting less than six weeks, data for symptomatic remission were available for over 900 patients in four trials (Table 3), giving a relative risk of 1.7 (1.4 to 2.0), and an NNT of 5.4 (4.0 to 8.1). At 6 to 12 weeks, in over 700 patients in three trials (Table 3), the relative risk was 1.5 (1.3 to 1.7) and the NNT 4.0 (3.1 to 5.5). All atypical antipsychotics appeared to perform equally well.

In active controlled trials there was no significant difference between treatments in trials shorter than six weeks (Table 4). In trials lasting 6–12 weeks remission rates with atypical antipsychotics (54%) were barely different than those with active control (48%) (Table 4).

Three trials reported on median time to remission. It was shorter for olanzapine than divalproex (14 vs 62 days; [27,45]), shorter for olanzapine plus mood stabiliser than placebo plus mood stabiliser (14 vs 22 days; [39]), but similar for olanzapine and haloperidol (34 vs 29 days; [41]).

Trials lasting longer than 12 weeks enrolled patients who had already responded to treatment with a lessening of symptoms, and so remission was not an outcome measured or reported in these trials.

#### Emergence of depression

Emergence of depression was generally defined as MADRS score of ≥18 with increase ≥4 from baseline on two consecutive occasions or at endpoint, or HAM-D score ≥15. Few trials lasting up to 12 weeks reported on the emergence of depression (Table 3). In placebo controlled trials lasting up to 12 weeks, no trial individually reported a significant difference, nor was there a difference when trials were combined.

**Figure 4:**
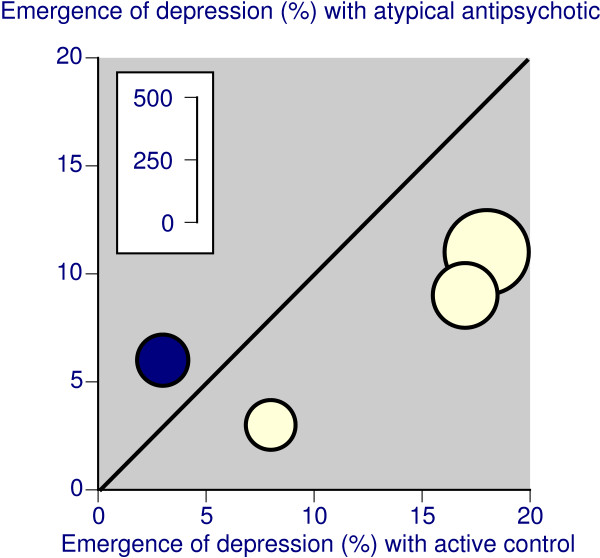
Emergence of depression with atypical antipsychotic or active comparator in placebo-controlled trials lasting 6–12 weeks, where the presenting episode was mania or mixed. The dark symbol indicates lithium as the comparator, and the light symbols haloperidol. The inset scale relates the number of patients in the comparison.

None of four active controlled trials lasting 6 to 12 weeks that reported this outcome individually reported significant difference between atypical antipsychotic and haloperidol or lithium. However, when combined, these trials with 1,000 patients reported significantly lower rates of emergence of depression with atypical antipsychotic (8%) than with active controls 13%) (Table 4), with a relative risk of 0.6 (0.4 to 0.9), and a NNT to prevent one emergent depression compared with active control of 21 (12 to 99). The active controls in this comparison were haloperidol and lithium, and atypical antipsychotics appeared to be particularly better than haloperidol (Figure 4). In the comparison with haloperidol alone in three trials and 795 patients, the NNT to prevent one emergent depression compared with haloperidol was 15 (9 to 48).

#### Relapse in maintenance trials

Trials lasting longer than 12 weeks were designed to investigate maintenance of remission, in terms of relapse into an affective state. They were therefore were much longer than 12 weeks; the range was 26 to 78 weeks.

In three placebo-controlled trials, with 589 patients [46,48,49], 135/332 (41%) suffered any relapse (depressive, manic or mixed) with atypical antipsychotic (olanzapine or aripiprazole), compared with 166/257 (65%) with placebo. The relative risk of relapse was 0.6 (0.5 to 0.7), with an NNTp to prevent a relapse of 4.2 (3.1 to 6.2) for olanzapine compared to placebo. Two active controlled trials (487 patients) had slightly lower relapse rates with olanzapine (32%) than lithium or divalproex (41%) but the difference was barely significant with a relative risk of 0.8 (0.6 to 0.98). Time to any relapse was longer for olanzapine than placebo (174 vs 22 days; [46]) and for olanzapine plus mood stabiliser than placebo plus mood stabiliser (163 vs 42 days; [46]), but there was no significant difference for olanzapine and lithium [47].

For relapse into a depressive state, there was bare significant difference (upper limit of confidence interval 0.98; Table 5) between atypical antipsychotic (25%), mostly olanzapine, and placebo (31%) in three trials with 589 patients [46,48,49] (relative risk 0.8; 95% confidence interval 0.6 to 0.98). For relapse into a manic state, there was a significant difference between atypical antipsychotic (12%), mostly olanzapine, and placebo (29%) in three trials with 589 patients [46,48,49], with a relative risk 0.4 (0.3 to 0.6) and NNTp to prevent one manic relapse of 5.9 (4.2 to 9.5).

**Table 5:** Outcomes in maintenance trials in bipolar mania – efficacy and discontinuations in trials lasting 26 weeks or longer [vs placebo 46. 48, 49 versus active 45, 47]

		**Events/total patients**		**Event rate (%)**			
				
**Outcome**	**Trials**	**Treatment**	**Active control**	**Treatment**	**Active control**	**Relative Risk (95% CI)**	**NNTp or NNH (95% CI)**
**Relapse**							
							
**Any relapse**							
All versus placebo	3	135/332	166/257	41	65	0.6 (0.5 to 0.7)	**4.2 (3.1 to 6.2)**
Olanzapine versus placebo	2	116/255	130/174	45	75	0.6 (0.5 to 0.7)	**3.4 (2.6 to 4.9)**
Olanzapine versus active	2	79/250	96/237	32	41	0.8 (0.6 to 0.98)	**11 (5.7 to 250)**
**Depressive relapse**							
All versus placebo	3	84/332	79/257	25	31	0.8 (0.6 to 0.98)	**18 (7.8 to 18)**
Olanzapine versus placebo	2	75/255	68/174	29	39	0.7 (0.6 to 0.97)	**10 (5.3 to 200)**
**Manic relapse**							
All versus placebo	3	39/332	74/257	12	29	0.4 (0.3 to 0.6)	**5.9 (4.2 to 9.5)**
Olanzapine versus placebo	2	33/255	55/174	13	32	0.4 (0.3 to 0.6)	**5.4 (3.7 to 9.4)**

**Discontinuation**							
							
**All cause**							
All versus placebo	3	146/354	116/267	41	43	1.1 (0.9 to 1.3)	not calculated
Olanzapine versus placebo	2	107/276	61/184	39	33	1.3 (1.04 to 1.7)	18 (6.9 to 30)
Olanzapine versus active	2	222/342	250/341	65	73	0.9 (0.8 to 0.98)	**12 (6.5 to 67)**
**Lack of efficacy**							
All versus placebo	3	36/354	55/267	10	21	0.6 (0.5 to 0.9)	**9.6 (6.2 to 22)**
Olanzapine versus placebo	2	17/276	19/184	6	10	0.8 (0.4 to 1.4)	not calculated
Olanzapine versus active	2	55/342	62/340	16	18	0.9 (0.6 to 1.2)	not calculated
**Adverse events**							
All versus placebo	3	27/354	9/267	8	4	2.4 (1.1 to 5.0)	24 (13 to 160)
Olanzapine versus placebo	2	22/276	8/184	8	5	2.0 (0.9 to 4.5)	not calculated
Olanzapine versus active	2	72/342	80/340	21	24	0.9 (0.7 to 1.2)	not calculated

For discontinuations NNTp is shown in **bold**, indicating fewer events with treatment than placebo. More events with treatment than placebo, NNH, is in plain text. Active comparators were haloperidol, lithium, or divalproex

#### Discontinuations

All cause discontinuations were less frequent with atypical antipsychotic than placebo in trials lasting less than six weeks (Table 3), and 6–12 weeks (Table 3). Discontinuations for lack of efficacy were also less common with atypical antipsychotic than placebo in trials lasting less than six weeks and 6–12 weeks, with a similar NNTp as for all cause discontinuations (Table 3). Discontinuations due to adverse events were not significantly different from placebo in trials lasting less than six weeks, but more common in trials lasting 6–12 weeks (Table 3).

In active controlled trials discontinuations for any cause, lack of efficacy or adverse events were not statistically different between treatments for trials lasting less than six weeks, but in trials lasting 6–12 weeks, both all cause and adverse event discontinuations were less common with atypical antipsychotic than active control (Table 4).

In long-term maintenance trials olanzapine had more all cause discontinuations than placebo, but fewer than active comparators, though event rates differed considerably (Table 5). Lack of efficacy and adverse event discontinuations did not differ significantly.

## Adverse events

Some adverse events were measured using a scale with predefined criteria; these include weight gain >7%, extrapyramidal symptoms, and glucose and cholesterol levels [see Additional files 1 and 2]. Other adverse events were spontaneously reported by patients or elicited by questions. Most trials reported events only if they occurred in at least 10% of any treatment group, although occasionally there was no lower limit or the limit was 5%, or events were reported if there was a statistically significant difference between groups [see Additional file 3].

Adverse events could be included in both categories. For instance, weight gain may have been measured and gains in excess of 7% reported as a pre-defined outcome, but weight gain may also have been reported by patients as an adverse event. Again, extrapyramidal symptoms may have been prospectively assessed, but patients may also have reported tremor or other symptoms.

Trials presenting with a manic, mixed or depressive episode were analysed together. Treatment emergent adverse events were frequently described as mild or moderate, or of limited duration, particularly somnolence or gastrointestinal events. Few trials specifically reported the absence of serious adverse events, or serious adverse events as a separate category.

### Measured adverse events

#### Weight gain >7% baseline

Predictably, short term trials lasting less than six weeks found no significant difference between atypical antipsychotic (quetiapine, aripiprazole or ziprasidone) and placebo (Table 6). In trials lasting 6–12 weeks [20,43,45] and over 12 weeks [46,48,49] significantly more patients gained this amount of weight with quetiapine, olanzapine and aripiprazole than with placebo.

**Table 6:** Adverse events in placebo controlled trials

		**Events/total patients**		**Event rate (%)**			
				
**Event**	**Trials**	**Treatment**	**Placebo**	**Treatment**	**Placebo**	**Relative Risk (95% CI)**	**NNH/NNTp (95% CI)**
**Less than 6 weeks [24, 26, 28, 30-35 37, 38]**							
Measured weight increase >7%	4	12/469	8/408	3	2	1.14 (0.5 to 2.8)	not calculated
Treatment emergent weight gain	2	20/266	6/272	8	2	3.4 (1.4 to 8.4)	19 (11 to 59)
Treatment emergent akathisia	5	73/738	26/599	10	4	2.2 (1.4 to 3.5)	18 (12 to 35)
Treatment emergent extrapyramidal disorder	5	115/566	30/477	20	6	3.5 (2.4 to 5.1)	7.1 (5.6 to 10)
Treatment emergent tremor	5	46/618	16/529	7	3	2.5 (1.4 to 4.3)	23 (14 to 53)
Treatment emergent somnolence	11	340/1293	96/1244	26	8	3.5 (2.8 to 4.3)	5.4 (4.7 to 6.4)
Treatment emergent depression	2	10/145	10/144	7	7	1.0 (0.4 to 2.3)	not calculated

**6–12 weeks [20, 22, 39, 41-43]**							
Measured weight increase >7%	4	136/1151	15/803	12	2	6.4 (3.9 to 11)	10 (8.3 to 13)
Treatment emergent weight gain	2	76/336	9/212	23	4	4.7 (2.5 to 9.1)	5.4 (4.2 to 7.6)
Treatment emergent akathisia	4	7/209	12/198	3	6	0.6 (0.2 to 1.4)	not calculated
Treatment emergent extrapyramidal disorder	3	79/800	31/448	10	7	1.6 (1.04 to 2.4)	33 (16 to infinity)
Treatment emergent tremor	3	67/438	25/313	15	8	1.6 (1.1 to 2.5)	14 (8.5 to 36)
Treatment emergent somnolence	6	450/1487	109/1015	30	11	2.7 (2.2 to 3.3)	5.1 (4.4 to 6.1)
Treatment emergent depression	3	49/438	25/313	11	8	1.1 (0.7 to 1.7)	not calculated

**More than 12 weeks [46, 48, 49]**							
Measured weight increase >7%	3	57/332	6/244	17	2	6.6 (3.0 to 15)	6.8 (5.2 to 9.9)
Treatment emergent weight gain	2	28/276	5/184	10	3	4.2 (1.6 to 11)	13 (9 to 32)
Treatment emergent somnolence	3	20/353	12/267	6	4	1.5 (0.7 to 3.0)	not calculated

NNTp is shown in **bold**, indicating fewer events with treatment than placebo. More events with treatment than placebo, NNH, is in plain text

In active controlled trials atypical antipsychotics olanzapine (and olanzapine and fluoxetine) and quetiapine produced more weight gain than active comparators over 6–12 weeks [21,43,45], as did olanzapine in trials lasting more than 12 weeks [45,47] (Table 7).

**Table 7:** Adverse events in active controlled trials

		**Events/total patients**		**Event rate (%)**			
				
**Event**	**Trials**	**Treatment**	**Active control**	**Treatment**	**Active control**	**Relative Risk (95% CI)**	**NNTp or NNH (95% CI)**
**Less than 6 weeks [27, 30, 37]**							
Treatment emergent tremor	3	24/331	26/323	7	8	0.9 (0.5 to 1.5)	not calculated
Treatment emergent somnolence	3	69/331	47/323	21	15	1.5 (1.1 to 2.0)	16 (8.3 to 210)

**6–12 weeks [21, 40, 41, 42, 43]**							
Measured weight increase >7%	3	152/536	39/515	31	13	3.6 (2.6 to 5.0)	4.8 (4.0 to 6.1)
Treatment emergent weight gain	3	62/398	21/380	16	6	2.9 (1.8 to 4.6)	10 (7.0 to 17)
Treatment emergent akathisia	4	42/618	110/585	7	19	0.4 (0.3 to 0.5)	**8.3 (6.4 to 12)**
Treatment emergent extrapyramidal disorder	3	27/511	148/490	5	30	0.2 (0.1 to 0.3)	**4.0 (3.4 to 4.9)**
Treatment emergent tremor	4	39/618	122/585	6	21	0.3 (0.2 to 0.4)	**6.9 (5.5 to 9.3)**
Treatment emergent somnolence	5	133/705	72/684	19	11	1.8 (1.4 to 2.4)	12 (8.3 to 22)

**More than 12 weeks [45, 47]**							
Measured weight increase >7%	2	93/340	43/337	27	13	2.2 (1.6 to 3.0)	6.9 (4.9 to 12)
Treatment emergent weight gain	2	45/342	25/340	13	7	1.8 (1.1 to 2.8)	17 (10 to 79)
Treatment emergent somnolence	2	64/342	31/340	19	9	2.1 (1.4 to 3.0)	10 (6.8 to 23)
Treatment emergent depression	2	88/342	63/340	26	19	1.4 (1.1 to 1.8)	14 (7.5 to 100)

NNTp is shown in **bold**, indicating fewer events with treatment than placebo. More events with treatment than placebo, NNH, is in plain text. Active comparators were haloperidol, lithium, or divalproex

#### Extrapyramidal symptoms

All trials assessed extrapyramidal symptoms using recognised scales (SAS, BARS, AIMS). Few reported actual numbers of patients affected, but rather reported lack of statistical difference [seeAdditional files 1 and 2]. Atypical antipsychotics were reported to produce symptoms in significantly fewer patients than haloperidol [37,41,43,44] and lithium [42].

#### Prolactin, glucose, lipids

There were few statistically significant changes in laboratory values, and no pattern of change with any treatment.

### Patient reported

Almost all trials did not report adverse events occurring below a frequency of 10%, with some occasionally using a lower threshold. In consequence, a number of adverse events were reported sporadically (like constipation, or nausea), making sensible analysis of them impossible.

#### Weight gain

In placebo-controlled trials of any duration above six weeks, treatment emergent weight gain was reported to occur at approximately the same rate as the rate of measured weight increase above 7% (Table 6) with both atypical antipsychotics (mainly olanzapine) and placebo. In active controlled trials, weight gain was reported as an adverse event less often than when it was measured as an outcome of the trial, both for atypical antipsychotic (mainly olanzapine) and active control (divalproex, lithium, and lamotrigine) (Table 7).

#### Extrapyramidal symptoms

In placebo-controlled trials the frequency of akathisia was higher than placebo in trials lasting less than six weeks, but not those lasting 6–12 weeks (Table 6). Tremor was more common in both. Where symptoms were reported as extrapyramidal disorder, information was available only for trials of less than six weeks, and in these short-term trials the rate of reporting (20%) for atypical antipsychotics (risperidone and ziprasidone) was significantly more common than with placebo (6%).

Compared with haloperidol and lithium, atypical antipsychotics (olanzapine, quetiapine, and aripiprazole) produced significantly lower rates of akathisia in trials of 6–12 weeks (Table 7). Tremor occurred at the same rate with atypical antipsychotics (olanzapine and risperidone) as with haloperidol and divalproex (Table 7) in trials of six weeks or less, but significantly less frequently for olanzapine, quetiapine and aripiprazole (6%) than haloperidol or lithium (21%) in trials lasting 6–12 weeks. In the single trial using lamotrigine as active comparator, tremor was reported at a higher rate with olanzapine plus fluoxetine [21].

#### Somnolence

Somnolence occurred significantly more often with atypical antipsychotics than placebo in trials lasting less than six weeks, or of 6–12 weeks (Table 6). In maintenance trials lasting longer than 12 weeks somnolence was not significantly different between atypical antipsychotic (olanzapine) and placebo, but with only 32 events reported in total, and at a much lower rate (6% with atypical) than in the shorter duration trials (26%–30%).

In active controlled trials somnolence was reported frequently with atypical antipsychotic in trials of less than 6 weeks (21%), 6–12 weeks (19%) and longer than 12 weeks (19%). It occurred more frequently than with active controls (haloperidol, divalproex, lithium, or lamotrigine) (Table 7).

#### Depression

Depression in mania trials did not occur more frequently with atypical antipsychotic than with placebo in trials of less than six weeks or of 6–12 weeks (Table 6). It was not reported in longer duration comparisons with placebo. In longer comparisons there was significantly more treatment emergent depression with olanzapine than divalproex and lithium (Table 7).

## Discussion

An evidence-based approach to therapy requires certain fundamentals in order to have confidence in a result, and most confidence comes from systematic review and meta-analysis of good quality randomised trials [50]. Trials should be free from known sources of bias, as far as is practically possible. This includes randomisation, blinding, and using an intention to treat population, or at least knowing about withdrawals and drop outs [51-53]. It also includes having information on sufficient numbers of patients [9].

This review extends that of Perlis et al, 2006 [54], which included trials published to 2004, with 18 trials, 4,304 patients in the treatment of mania. This review also includes depression, and included information from 27 trials published to end 2006 with 7,838 patients. In addition to pooling information from these trials on efficacy, as did Perlis et al, we have also pooled information of adverse events.

A number of other systematic reviews and meta-analyses have addressed similar topics. Two Cochrane reviews [55,56] report on olanzapine and risperidone in acute mania. Other reviews have concentrated on particular aspects – mania, for instance, [54,57,58], or bipolar depression [59], or maintenance [60]. While generally similar, they have tended to use different methods. For instance, most concentrated on continuous outcomes, but a problem with mean changes in rating scales is that they can often be mean results of highly skewed distributions, making the means meaningless [61]. This review concentrated on dichotomous outcomes reflecting clinically relevant endpoints of efficacy and harm, and included four atypical antipsychotics over the short, medium, and long term, with trials included if they were published up to December 2006. Different approaches can be helpful in a number of ways, perhaps principally in providing information in ways that can be used by a wider audience.

There is an additional point of contention in systematic reviews and meta-analysis that is relevant here, namely how much it is acceptable to combine data from similar but not identical interventions, participants, duration, or outcome. We have chosen what we believe is a sensible middle course. Efficacy data are shown by both combined and by individual drugs (while recognising that numbers for some outcomes for some drugs may be small). Adverse events are not well reported, and we have chosen to combine the data. Additional files have the results from individual trials, so that others may perform analyses based on their logic or preference.

Trial design and outcomes have also to be valid and useful. This means, for instance, that in life-long illness we have longer rather than shorter trials, or that trials study appropriate patients without unrealistic exclusions or inclusions. It also means that outcomes have to be clinically relevant, and measured and reported in ways that are useful. For instance, a mean change in a composite measure is less useful than knowing the number of patients who have achieved an adequate level of response. Clinical trials reported in journals are limited in the amount they can report, and we know that using more detailed clinical trial reports improves both data access and utility [62-64].

The trials included in this review were of good reporting quality. All but one of the included trials scored 3 points or more out of a maximum of 5, a level known to limit the possibility of bias [52]. Points were lost due to inadequate descriptions of randomisation/allocation or blinding methods, and it was likely that allocation and blinding was, in fact, better than reported.

Trials were disparate in terms of atypical antipsychotic used, with olanzapine and quetiapine most commonly used in depression and in mania studies of less than 12 weeks, and olanzapine and aripiprazole the only atypicals tested in long-term maintenance studies. On the one hand, dividing studies by type of presenting episode, by duration, and by comparator, meant limiting the number of patients in each group available for analysis. On the other, combining these trials meant introducing a potentially unacceptable level of clinical heterogeneity. We chose to avoid this as much as possible by analysing by presenting episode and duration, but combining different atypical antipsychotics with a common comparator (placebo or active). There are potential problems with this approach, exemplified by apparent differences in efficacy between olanzapine and quetiapine in bipolar depression (Table 2).

Reporting of outcomes in trials was limiting. Although the number of patients experiencing response and remission were reported, form many other efficacy outcomes like depression and mania rating scores, or global impression, were predominantly reported as mean changes only, when it would be more useful to know how many patients experienced clinically relevant outcomes. The clinical relevance of some efficacy outcomes has also been challenged. For instance, re-analysis of an open-label extension of a randomised trial suggested a better outcome of sustained clinical recovery, where remission was sustained for at least eight weeks, rather than just occurring for any duration [65]. This outcome was not reported in any trial included in the review, and if used would give a much lower, but perhaps more realistic, impression of efficacy.

Trials usually only reported adverse events occurring in at least 10% of patients, so that for many events only sporadic information was available [see Additional file 3], and no analysis of adverse events could be complete. In addition, adverse event reporting could overlap. For example extrapyramidal symptoms like tremor or akathisia might be reported by patients alongside extrapyramidal syndrome or symptoms measured using recognised scales. Moreover, most trials permitted use of medication to treat extrapyramidal symptoms when they occurred, which could, of course, result in lower scores than otherwise on symptom rating scales. Extrapyramidal symptoms would still be recorded as spontaneous adverse events. Because patients experiencing extrapyramidal symptoms are more likely to withdraw or require dose reduction, it is possible that use of anticholinergics may have affected attrition rates, though related better to clinical practice.

The evidence available allows a number of inferences. Where the presenting episode was depression, both olanzapine and quetiapine appear to be efficacious over eight weeks, with more responses and remissions than placebo, and fewer lack of efficacy withdrawals. Adverse event withdrawals were higher than with placebo. There is some evidence that quetiapine at target doses of 300 or 600 mg daily is more efficacious than olanzapine, but at the cost of more adverse event discontinuations.

Where the presenting episode was mania, olanzapine, risperidone, and quetiapine had similar event rates and NNTs compared with placebo in trials shorter than six weeks (Table 3). Combining all atypicals compared with placebo, NNTs for response and remission were about 5. In trials lasting 6–12 weeks, NNTs for response or remission were somewhat better, at about 4. This is generally in accord with a previous meta-analysis [54], though that review combined continuous data to come to the conclusion that atypical antipsychotics were superior to placebo. Atypical antipsychotics produced fewer discontinuations for any cause or lack of efficacy, but somewhat more adverse event discontinuations in longer studies.

Limited comparison with active controls (lithium, valproate, haloperidol, lamotrigine) showed that there was no major difference in efficacy or discontinuations, in shorter duration trials. Perhaps notable, though, was a significantly reduced rate of adverse event discontinuations for atypicals than older active comparators in trials over 6–12 weeks.

## Conclusion

In general, atypical antipsychotics are effective in treating both phases of bipolar disorder compared with placebo, and as effective as established drug therapies, though only two (olanzapine and quetiapine) have been tested where the presenting episode was depression. In general, atypical antipsychotics produce fewer extrapyramidal symptoms, but weight gain is more common with olanzapine and quetiapine. There is insufficient data to confidently distinguish between different atypical antipsychotics, predominantly due to the clinical heterogeneity engendered by presentation, drug, dose, comparator, duration of trial, and outcomes measured. Moreover, the weight of evidence of efficacy in bipolar depression resides with olanzapine and quetiapine, and that on weight gain overwhelmingly with olanzapine; extrapolation to other drugs in the class may not be appropriate in these circumstances.

## Competing interests

The author(s) declare that they have no competing interests.

## Authors' contributions

RAM and SD were involved with the original concept, planning the study, data extraction, analysis, and preparing a manuscript. Both authors read and approved the final manuscript. Study design, methods, decision about study inclusion, analysis, and writing were the sole responsibility of the authors.

## Pre-publication history

The pre-publication history for this paper can be accessed here:



## Supplementary Material

Additional file 1 Trials of atypical antipsychotics in depression – design, details, and outcomes. A listing of the trials, with their main design features, efficacy outcomes, discontinuations, and adverse event reporting. Click here for file

Additional file 2 Trials of atypical antipsychotics in mania/mixed states – design, details, and outcomes. A listing of the trials, with their main design features, efficacy outcomes, discontinuations, and adverse event reporting. Click here for file

Additional file 3 Individual adverse events in all trials. A listing of the individual adverse events reported in the trials. Click here for file

Additional file 4 Excluded papers. Papers excluded, with reason for exclusion. Click here for file

## References

[B1] Swann AC (2006). What is bipolar disorder?. Am J Psychiatry.

[B2] Schaffer A, Cairney J, Cheung A, Veldhuizen S, Levitt A (2006). Community survey of bipolar disorder in Canada: lifetime prevalence and illness characteristics. Can J Psychiatry.

[B3] Grant BF, Stinson FS, Hasin DS, Dawson DA, Chou SP, Ruan WJ, Huang B (2005). Prevalence, correlates, and comorbidity of bipolar I disorder and axis I and II disorders: results from the National Epidemiologic Survey on Alcohol and Related Conditions. J Clin Psychiatry.

[B4] Perlis RH, Ostacher MJ, Patel JK, Marangell LB, Zhang H, Wisniewski SR, Ketter TA, Miklowitz DJ, Otto MW, Gyulai L, Reilly-Harrington NA, Nierenberg AA, Sachs GS, Thase ME (2006). Predictors of recurrence in bipolar disorder: primary outcomes from the Systematic Treatment Enhancement Program for Bipolar Disorder (STEP-BD). Am J Psychiatry.

[B5] Bagnall AM, Jones L, Ginnelly L, Lewis R, Glanville J, Gilbody S, Davies L, Torgerson D, Kleijnen J (2003). A systematic review of atypical antipsychotic drugs in schizophrenia. Health Technol Assess.

[B6] Lee PE, Gill SS, Freedman M, Bronskill SE, Hillmer MP, Rochon PA (2004). Atypical antipsychotic drugs in the treatment of behavioural and psychological symptoms of dementia: systematic review. BMJ.

[B7] Jadad AR, Moore RA, Carroll D, Jenkinson C, Reynolds DJ, Gavaghan DJ, McQuay HJ (1996). Assessing the quality of reports of randomized clinical trials: is blinding necessary?. Control Clin Trials.

[B8] Moher D, Cook DJ, Eastwood S, Olkin I, Rennie D, Stroup DF (1999). Improving the quality of reports of meta-analyses of randomised controlled trials: the QUOROM statement. Quality of Reporting of Meta-analyses. Lancet.

[B9] Moore RA, Gavaghan D, Tramer MR, Collins SL, McQuay HJ (1998). Size is everything – large amounts of information are needed to overcome random effects in estimating direction and magnitude of treatment effects. Pain.

[B10] Gavaghan DJ, Moore RA, McQuay HJ (2000). An evaluation of homogeneity tests in meta-analyses in pain using simulations of individual patient data. Pain.

[B11] Sterne JA, Gavaghan D, Egger M (2000). Publication and related bias in meta-analysis: power of statistical tests and prevalence in the literature. J Clin Epidemiol.

[B12] Terrin N, Schmid CH, Lau J (2005). In an empirical evaluation of the funnel plot, researchers could not visually identify publication bias. J Clin Epidemiol.

[B13] L'Abbe KA, Detsky AS, O'Rourke K (1987). Meta-analysis in clinical research. Ann Intern Med.

[B14] Morris JA, Gardner MJ, Gardner MJ, Altman DG (1995). Calculating confidence intervals for relative risk, odds ratios and standardised ratios and rates. Statistics with confidence – confidence intervals and statistical guidelines.

[B15] Cook RJ, Sackett DL (1995). The number needed to treat: a clinically useful measure of treatment effect. BMJ.

[B16] Tramèr MR, Reynolds DJM, Moore RA, McQuay HJ (1997). Impact of covert duplicate publication on meta-analysis: a case study. British Medical Journal.

[B17] Moore RA, Derry S, Makinson GT, McQuay HJ (2005). Tolerability and adverse events in clinical trials of celecoxib in osteoarthritis and rheumatoid arthritis: systematic review and meta-analysis of information from company clinical trial reports. Arthritis Res Ther.

[B18] Tohen M, Vieta E, Calabrese J, Ketter TA, Sachs G, Bowden C, Mitchell PB, Centorrino F, Risser R, Baker RW (2003). Efficacy of olanzapine and olanzapine-fluoxetine combination in the treatment of bipolar I depression. Arch Gen Psychiatry.

[B19] Shelton RC, Stahl SM (2004). Risperidone and paroxetine given singly and in combination for bipolar depression. J Clin Psychiatry.

[B20] Calabrese JR, Keck PE, Macfadden W, Minkwitz M, Ketter TA, Weisler RH, Cutler AJ, McCoy R, Wilson E, Mullen J (2005). A randomized, double-blind, placebo-controlled trial of quetiapine in the treatment of bipolar I or II depression. Am J Psychiatry.

[B21] Brown EB, McElroy SL, Keck PE, Deldar A, Adams DH, Tohen M, Williamson DJ (2006). A 7-week, randomized, double-blind trial of olanzapine/fluoxetine combination versus lamotrigine in the treatment of bipolar I depression. J Clin Psychiatry.

[B22] Thase ME, Macfadden W, Weisler RH, Chang W, Paulsson B, Khan A, Calabrese JR, BOLDER II Study Group (2006). Efficacy of quetiapine monotherapy in bipolar I and II depression: a double-blind, placebo-controlled study (the BOLDER II study). J Clin Psychopharmacol.

[B23] Segal J, Berk M, Brook S (1998). Risperidone compared with both lithium and haloperidol in mania: a double-blind, randomized controlled trial. Clin Neuropharmacol.

[B24] Tohen M, Sanger TM, McElroy SL, Tollefson GD, Chengappa KN, Daniel DG, Petty F, Centorrino F, Wang R, Grundy SL (1999). Olanzapine versus placebo in the treatment of acute mania. Olanzapine HGEH Study Group. Am J Psychiatry.

[B25] Berk M, Ichim L, Brook S (1999). Olanzapine compared to lithium in mania: a double-blind randomized controlled trial. Int Clin Psychopharmacol.

[B26] Tohen M, Jacobs TG, Grundy SL, McElroy SL, Banov MC, Janicak PG, Sanger T, Risser R, Zhang F, Toma V (2000). Efficacy of olanzapine in acute bipolar mania: a double-blind, placebo-controlled study. The Olanzipine HGGW Study Group. Arch Gen Psychiatry.

[B27] Tohen M, Baker RW, Altshuler LL, Zarate CA, Suppes T, Ketter TA, Milton DR, Risser R, Gilmore JA, Breier A (2002). Olanzapine versus divalproex in the treatment of acute mania. Am J Psychiatry.

[B28] Hirschfeld RM, Keck PE, Kramer M, Karcher K, Canuso C, Eerdekens M, Grossman F (2004). Rapid antimanic effect of risperidone monotherapy: a 3-week multicenter, double-blind, placebo-controlled trial. Am J Psychiatry.

[B29] Yatham LN, Grossman F, Augustyns I, Vieta E, Ravindran A (2003). Mood stabilisers plus risperidone or placebo in the treatment of acute mania. International, double-blind, randomised controlled trial. Br J Psychiatry.

[B30] Sachs GS, Grossman F, Ghaemi SN, Okamoto A, Bowden CL (2002). Combination of a mood stabilizer with risperidone or haloperidol for treatment of acute mania: a double-blind, placebo-controlled comparison of efficacy and safety. Am J Psychiatry.

[B31] Sachs G, Chengappa KN, Suppes T, Mullen JA, Brecher M, Devine NA, Sweitzer DE (2004). Quetiapine with lithium or divalproex for the treatment of bipolar mania: a randomized, double-blind, placebo-controlled study. Bipolar Disord.

[B32] Yatham LN, Paulsson B, Mullen J, Vagero AM (2004). Quetiapine versus placebo in combination with lithium or divalproex for the treatment of bipolar mania. J Clin Psychopharmacol.

[B33] Keck PE, Versiani M, Potkin S, West SA, Giller E, Ice K (2003). Ziprasidone in the treatment of acute bipolar mania: a three-week, placebo-controlled, double-blind, randomized trial. Am J Psychiatry.

[B34] Keck PE, Marcus R, Tourkodimitris S, Ali M, Liebeskind A, Saha A, Ingenito G (2003). A placebo-controlled, double-blind study of the efficacy and safety of aripiprazole in patients with acute bipolar mania. Am J Psychiatry.

[B35] Potkin SG, Keck PE, Segal S, Ice K, English P (2005). Ziprasidone in acute bipolar mania: a 21-day randomized, double-blind, placebo-controlled replication trial. J Clin Psychopharmacol.

[B36] Khanna S, Vieta E, Lyons B, Grossman F, Eerdekens M, Kramer M (2005). Risperidone in the treatment of acute mania: double-blind, placebo-controlled study. Br J Psychiatry.

[B37] Smulevich AB, Khanna S, Eerdekens M, Karcher K, Kramer M, Grossman F (2005). Acute and continuation risperidone monotherapy in bipolar mania: a 3-week placebo-controlled trial followed by a 9-week double-blind trial of risperidone and haloperidol. Eur Neuropsychopharmacol.

[B38] Sachs G, Sanchez R, Marcus R, Stock E, McQuade R, Carson W, Abou-Gharbia N, Impellizzeri C, Kaplita S, Rollin L, Iwamoto T, Aripiprazole Study Group (2006). Aripiprazole in the treatment of acute manic or mixed episodes in patients with bipolar I disorder: a 3-week placebo-controlled study. J Psychopharmacol.

[B39] Tohen M, Chengappa KN, Suppes T, Zarate CA, Calabrese JR, Bowden CL, Sachs GS, Kupfer DJ, Baker RW, Risser RC (2002). Efficacy of olanzapine in combination with valproate or lithium in the treatment of mania in patients partially nonresponsive to valproate or lithium monotherapy. Arch Gen Psychiatry.

[B40] Zajecka JM, Weisler R, Sachs G, Swann AC, Wozniak P, Sommerville KW (2002). A comparison of the efficacy, safety, and tolerability of divalproex sodium and olanzapine in the treatment of bipolar disorder. J Clin Psychiatry.

[B41] Tohen M, Goldberg JF, Gonzalez-Pinto Arrillaga AM, Azorin JM, Vieta E, Hardy-Bayle MC, Lawson WB, Emsley RA, Zhang F, Baker RW (2003). A 12-week, double-blind comparison of olanzapine vs haloperidol in the treatment of acute mania. Arch Gen Psychiatry.

[B42] Bowden CL, Grunze H, Mullen J, Brecher M, Paulsson B, Jones M, Vagero M, Svensson K (2005). A randomized, double-blind, placebo-controlled efficacy and safety study of quetiapine or lithium as monotherapy for mania in bipolar disorder. J Clin Psychiatry.

[B43] McIntyre RS, Brecher M, Paulsson B, Huizar K, Mullen J (2005). Quetiapine or haloperidol as monotherapy for bipolar mania – a 12-week, double-blind, randomised, parallel-group, placebo-controlled trial. Eur Neuropsychopharmacol.

[B44] Vieta E, Bourin M, Sanchez R, Marcus R, Stock E, McQuade R, Carson W, Abou-Gharbia N, Swanink R, Iwamoto T (2005). Effectiveness of aripiprazole v. haloperidol in acute bipolar mania: double-blind, randomised, comparative 12-week trial. Br J Psychiatry.

[B45] Tohen M, Ketter TA, Zarate CA, Suppes T, Frye M, Altshuler L, Zajecka J, Schuh LM, Risser RC, Brown E (2003). Olanzapine versus divalproex sodium for the treatment of acute mania and maintenance of remission: a 47-week study. Am J Psychiatry.

[B46] Tohen M, Chengappa KN, Suppes T, Baker RW, Zarate CA, Bowden CL, Sachs GS, Kupfer DJ, Ghaemi SN, Feldman PD (2004). Relapse prevention in bipolar I disorder: 18-month comparison of olanzapine plus mood stabiliser v. mood stabiliser alone. Br J Psychiatry.

[B47] Tohen M, Greil W, Calabrese JR, Sachs GS, Yatham LN, Oerlinghausen BM, Koukopoulos A, Cassano GB, Grunze H, Licht RW (2005). Olanzapine versus lithium in the maintenance treatment of bipolar disorder: a 12-month, randomized, double-blind, controlled clinical trial. Am J Psychiatry.

[B48] Tohen M, Calabrese JR, Sachs GS, Banov MD, Detke HC, Risser R, Baker RW, Chou JC, Bowden CL (2006). Randomized, placebo-controlled trial of olanzapine as maintenance therapy in patients with bipolar I disorder responding to acute treatment with olanzapine. Am J Psychiatry.

[B49] Keck PE, Calabrese JR, McQuade RD, Carson WH, Carlson BX, Rollin LM, Marcus RN, Sanchez R, Aripiprazole Study Group (2006). A randomized, double-blind, placebo-controlled 26-week trial of aripiprazole in recently manic patients with bipolar I disorder. J Clin Psychiatry.

[B50] Ioannidis JP (2005). Why most published research findings are false. PLoS Med.

[B51] Moher D, Pham B, Jones A, Cook DJ, Jadad AR, Moher M, Tugwell P, Klassen TP (1998). Does quality of reports of randomised trials affect estimates of intervention efficacy reported in meta-analyses?. Lancet.

[B52] Khan KS, Daya S, Jadad A (1996). The importance of quality of primary studies in producing unbiased systematic reviews. Arch Intern Med.

[B53] Schulz KF (1995). Subverting randomization in controlled trials. JAMA.

[B54] Perlis RH, Welge JA, Vornik LA, Hirschfeld RM, Keck PE (2006). Atypical antipsychotics in the treatment of mania: a meta-analysis of randomized, placebo-controlled trials. J Clin Psychiatry.

[B55] Rendell JM, Gijsman HJ, Keck P, Goodwin GM, Geddes JR (2003). Olanzapine alone or in combination for acute mainia. Cochrane Database Syst Rev.

[B56] Rendell JM, Gijsman HJ, Bauer MS, Goodwin GM, Geddes GR (2006). Risperidone alone or in combination for acute mania. Cochrane Database Syst Rev.

[B57] Smith LA, Cornelius V, Warnock A, Tacchi MJ, Taylor D (2007). Acute bipolar mania: a systematic review and meta-analysis of co-therapy vs. monotherapy. Acta Psychiatr Scand.

[B58] Scherk H, Pajonk FG, Leucht S (2007). Second-generation antipsychotic agents in the treatment of acute mania: a systematic review and meta-analysis of randomized controlled trials. Arch Gen Psychiatry.

[B59] Gao K, Gajwani P, Elhaj O, Calabrese JR (2005). Typical and atypical antipsychotics in bipolar depression. J Clin Psychiatry.

[B60] Muzina DJ, Calabrese JR (2005). Maintenance therapies in bipolar disorder: focus on randomized controlled trials. Aust N Z J Psychiatry.

[B61] McQuay H, Carroll D, Moore A (1996). Variation in the placebo effect in randomised controlled trials of analgesics: all is as blind as it seems. Pain.

[B62] Moore RA, Edwards JE, McQuay HJ (2002). Sildenafil (Viagra) for male erectile dysfunction: a meta-analysis of clinical trial reports. BMC Urol.

[B63] Edwards JE, McQuay HJ, Moore RA (2004). Efficacy and safety of valdecoxib for treatment of osteoarthritis and rheumatoid arthritis: systematic review of randomised controlled trials. Pain.

[B64] Moore RA, Derry S, Makinson GT, McQuay HJ (2005). Tolerability and adverse events in clinical trials of celecoxib in osteoarthritis and rheumatoid arthritis: systematic review and meta-analysis of information from company clinical trial reports. Arthritis Res Ther.

[B65] Chengappa KN, Hennen J, Baldessarini RJ, Kupfer DJ, Yatham LN, Gershon S, Baker RW, Tohen M (2005). Recovery and functional outcomes following olanzapine treatment for bipolar I mania. Bipolar Disord.

