# Supplementary material for: Atypical antipsychotics in bipolar disorder: systematic review of randomised trials
Source: BMC Psychiatry. 2007 Aug 16;7:40. doi: 10.1186/1471-244X-7-40 (PMC2020469; doi:10.1186/1471-244X-7-40)
Supplement: Additional file 1 — Trials of atypical antipsychotics in depression – design, details, and outcomes. A listing of the trials, with their main design features, efficacy outcomes, discontinuations, and adverse event reporting. [file 1471-244X-7-40-S1.pdf]

Additional file 1: Trials of atypical antipsychotics in depression - design, details, and outcomes

										Discontinuations				Adverse event reporting				Scale-defined adverse events	
**Reference [number]**	**Patients**	**Treatment**	**Mean daily dose Other medication**	**Duration (weeks)**	**QS**	**Response**	**Remission**	**CGI**	**Emergence of mania**	**All cause**	**Lack of efficacy**	**Adverse event**	**T-E mania**	**Patients experiencing any adverse event**	**Severe**	**Serious**	**Treatment related**	**EP symptoms**	**Weight gain ≥7%**
Tohen et al. 2003<br>[18]	Bipolar I depression, (MADRS >19)<br>Age:42 ±12 years<br>M:47%<br>Psychotic: 12.5%<br>Rapid cycling: 37%	(1) olanzapine 5-20 mg/day, n=351<br>(2) olanzapine-fluoxetine 6/25, 6/50 or 12/50 mg/day, n=82<br>(3) placebo, n=355	(1) 9.7 mg<br>(2) 7.4 mg/39.4 mg<br><br>Benzodiazepines (36-44%)<br>Anticholinergics to treat EP sympt (3-8%)	8	R2, D1, W1	≥50% improvement in MADRS, min 4 wks<br>(1) 137/351<br>(2) 46/82<br>(3) 108/355<br>Median time to response (days):<br>(1) 55<br>(2) 21<br>(3) 59	MADRS ≤12, min 4 wks<br>(1) 115/351<br>(2) 40/82<br>(3) 87/355<br>Median time to remission(days):<br>(1) 57<br>(2) 42<br>(3) 59	no categorical data<br>Mean improvements sig greater for (2) than (1) than (3)	YMRS <15 at baseline and ≥15 any time thereafter<br>(1) 19/335<br>(2) 5/78<br>(3) 23/345	(1) 191/351<br>(2) 31/82<br>(3) 232/355	(1) 73/351<br>(2) 8/82<br>(3) 121/355	(1) 34/351<br>(2) 2/82<br>(3) 19/355	(1) 15/351<br>(2) 4/82<br>(3) 24/355	no data	no data	no data	no data	SAS and AIMS<br>No sig diff	
Shelton, Stahl. 2004<br>[19]	Bipolar I and II disorder, depressive episode (HAM-D>17), receiving mood stabiliser<br>Age:36 ±10 years<br>M:50%<br>Psychotic: 0%	(1) risperidone 1-4 mg/day + placebo plus mood stabiliser, n=10<br>(2) paroxetine 20-40 mg/day + placeo plus mood stabiliser, n=10<br>(3) risperidone +paroxetine plus mood stabiliser, n=10	(1) 2.15 mg<br>(2) 35 mg<br>(3) 1.16 mg/22 mg<br><br>Lorazepam in first 4 weeks	12	R1, D2, W1	≥50% improvement on HAM-D and 1 or 2 on CGI-S (LOCF)<br>(1) 3/10<br>(2) 2/10<br>(3) 3/10	HAM-D ≤7 and no longer MDD using DSM-IV (LOCF)<br>(1) 1/10<br>(2) 2/10<br>(3) 3/10	no categorical data	none<br>(one pt in (2) experienced v mild hypomania)	(1) 5/10<br>(2) 2/10<br>(3) 4/10	(1) 3/10<br>(2) 0/10<br>(3) 1/10	(1) 1/10<br>(2) 1/10<br>(3) 3/10	none	no data	no data	no data	no data	SAS, BARS<br>No sig diff	
Calabrese et al. 2005<br>[20]	Bipolar I and II disorder, major depressive episode (HAM-D≥20, YMRS≤12)<br>Age:37 ±11 years<br>M:42%<br>Excl: history non-response to >2 classes antidepressants for current episode	(1) quetiapine 600 mg/day, n=170<br>(2) quetiapine 300 mg/day, n=172<br>(3) placebo, n=169<br>[ITT cohort]	N/A<br><br>Lorazepam and zolpidem during first 3 weeks - generally <10%	8	R2, D2, W1	≥50% improvement in MADRS<br>(1) 99/170<br>(2) 99/172<br>(3) 61/169<br>Median time to response(days):<br>(1) 22<br>(2) 22<br>(3) 36	MADRS ≤12<br>(1) 90/170<br>(2) 91/172<br>(3) 48/169<br>Median time to remission (days):<br>(1) 27<br>(2) 29<br>(3) 65	normal/not at all ill/borderline ill<br>(1) 72/170<br>(2) 66/172<br>(3) 40/169	YMRS ≥16 on 2 consec visits or at end point or AE of mania (using safety population)<br>(1) 7/179<br>(2) 7/180<br>(3) 7/180	(1) 82/180<br>(2) 60/181<br>(3) 74/181	(1) 1/180<br>(2) 4/181<br>(3) 24/181	(1) 47/180<br>(2) 29/181<br>(3) 15/181	no data	no data	no data	(1) 9/180<br>(2) 6/181<br>(3) 16/181<br>(none T-R)	no data	SAS, BARS: 9% to 15% with an inc from baseline, with no sig diff between groups	
Brown et al. 2006<br>[21]	Bipolar I disorder, depressive episode, (MADRS >19)<br>Age:37 ±11 years<br>M:40%<br>Psychotic: 6%<br>Rapid cycling: 34%<br>Excl: YMRS≥15, history non-response to study drugs	(1) olanzapine-fluoxetine 6/25, 12/25, 6/50 or 12/50 mg/day, n=205<br>(2) Lamotrigine150-200 mg/day, n=205	(1) 10.7/38.3 mg<br>(2) 106.4 mg<br><br>Benzodiazepines (22%)<br>Anticholinergics (1%)	7	R1, D1, W1	≥50% improvement in MADRS<br>(1) 141/205<br>(2) 122/205<br>Median time to response(days):<br>(1) 17<br>(2) 23	MADRS ≤12, at last observation<br>(1) 116/205<br>(2) 101/205<br>Median time to remission(days):<br>(1) 32<br>(2) 41	CGI-S ≤3<br>(1) 147/205<br>(2) 132/205<br><br>Mean improvement sig greater for (1) than (2)	YMRS>15 at any time<br>(1) 8/205<br>(2) 11/205	(1) 68/205<br>(2) 71/205	(1) 2/205<br>(2) 4/205	(1) 17/205<br>(2) 15/205	no data	no data	incomplete data<br>Suicide attempts:<br>(1) 1/205<br>(2) 2/205<br>All suicidal and self harm events:<br>(1) 1/205<br>(2) 7/205	no data	no data	AIMS: no sig diff between groups in severity of tardive dyskinesia	(1) 48/205<br>(2) 0/205
Thase et al. 2006<br>[22]	Bipolar I and II disorder, major depressive episode (HAM-D≥20, YMRS≤12)<br>Age:37 ±11 years<br>M:43%<br>Excl: history non-response to >2 classes antidepressants for current episode. Current episode <4 weeks or >12 months	(1) quetiapine 600 mg/day, n=151<br>(2) quetiapine 300 mg/day, n=155<br>(3) placebo, n=161<br>[ITT cohort]	N/A<br><br>Lorazepam and zolpidem during first 3 weeks - generally low, <4%	8	R1, D2, W1	≥50% improvement in MADRS<br>(1) 88/151<br>(2) 93/155<br>(3) 71/161<br><br>Sig diff between (1) or (2) and (3) by week 2	MADRS ≤12<br>(1) 79/151<br>(2) 80/155<br>(3) 48/161<br><br>Sig diff between (2) and (3) by week 2, and for (1) and (3) by week 3	normal/not at all ill/borderline ill<br>(1) 49/151<br>(2) 64/155<br>(3) 39/161<br>Much improved/ very much improved<br>(1) 91/151<br>(2) 95/155<br>(3) 62/161	YMRS ≥16 on 2 consec visits or at end point or AE of mania (using safety population)<br>(1) 6/168<br>(2) 3/171<br>(3) 11/167	(1) 79/169<br>(2) 71/172<br>(3) 58/168	(1) 5/169<br>(2) 3/172<br>(3) 13/168	(1) 19/169<br>(2) 14/172<br>(3) 2/168	no data	no data	no data	(1) 7/169<br>(2) 3/172<br>(3) 1/168<br>(none T-R)	no data	SAS, BARS<br>No sig change from baseline in any group	(1) 11/168<br>(2) 5/171<br>(3) 4/167

QS = quality score; R = randomised; D = double blind; W = withdrawals or drop outs; CGI = clinical global impression; TE = treatment emergent; EP = extrapyramidal; AIMS = abnormal involuntary movement scale; SAS = Simpson-Angus rating scale; BARS = Barnes akathisia rating scale
