# Supplementary material for: Atypical antipsychotics in bipolar disorder: systematic review of randomised trials
Source: BMC Psychiatry. 2007 Aug 16;7:40. doi: 10.1186/1471-244X-7-40 (PMC2020469; doi:10.1186/1471-244X-7-40)
Supplement: Additional file 2 — Trials of atypical antipsychotics in mania/mixed states – design, details, and outcomes. A listing of the trials, with their main design features, efficacy outcomes, discontinuations, and adverse event reporting. [file 1471-244X-7-40-S2.pdf]

Additional file 2: Trials of atypical antipsychotics in mania/mixed states - design, details, and outcomes

Reference [number]	Patients	Treatment	Median/modal dose	Concommitant medication	Duration (weeks)	QS	Response	Remission	CGI	Depression score	Symptomatic depression or affective episode
**Less than 6 weeks**											
**Segal et al. 1998<br>[23]**	**Bipolar disorder, manic<br>Age: 34 years<br>M: 22%**	**(1) risperidone 6 mg/day, n=15<br>(2) haloperidol 10 mg/day, n=15<br>(3) lithium to give 0.6-1.2mmol/L, n=15**	**no data**	**Benzodiazepines<br>Anticholinergics for EP symptoms**	**4**	**R1, D1, W0**	**mean data only**	**no data**	**mean changes small and did not differ between groups**	**no data**	**no data**
Tohen et al. 1999<br>[24]	Bipolar disorder, manic or mixed<br>Age:40±11 years<br>M: 52%<br>manic:83%<br>mixed:17%	(1) olanzapine 5-20 mg/day, n=70<br>(2) placebo, n=69	15 mg/day	Benzodiazepines (86%)<br>Anticholinergics for EP symptoms (9.4%)<br>No sig differences	3	R1, D1, W1	≥50% dec in YMRS at endpoint<br>(1) 34/70<br>(2) 16/69	no data	mean changes in CGI-BP scales given, no categorical data<br>Overall: no sig diff between olanzapine and placeo	no data	no data
Berk et al. 1999<br>[25]	Bipolar disorder, manic<br>Age: 31 years<br>M: 57%	(1) olanzapine 10 mg/day, n=15<br>(2) lithium 800 mg/day	no data	Benzodiazepines<br>Anticholinergics for EP symptoms	4	R1<br>D1<br>R1	mean data only	no data	mean changes in CGI-BP scales given, no categorical data<br>Overall: olanzapine sig better than lithium	no data	no data
Tohen et al. 2000<br>[26]	Bipolar disorder, manic or mixed<br>Age:39 years<br>M: 50%<br>manic:58%<br>mixed:42%	(1) olanzapine 5-20 mg/day, n=55<br>(2) placebo, n=60	20 mg/day	Benzodiazepines (70%)<br>Anticholinergics for EP symptoms (negligible)<br>No sig differences	4	R1, D1, W1	≥50% dec in YMRS at endpoint<br>(1) 35/55<br>(2) 25/60	Euthymic: YMRS ≤12<br>(1) 33/55<br>(2) 20/60	mean changes in CGI-BP scales given, no categorical data<br>Overall: olanzapine better than placeo	Mean values improved in both groups. Greater improvement seen for olanzapine in subset with HAMD-21 ≥20 at baseline	≥3 point worsening in HAMD-21<br>(1) 6/55<br>(2) 10/60
Tohen et al. 2002<br>See Tohen 2003b for 47 week study<br>[27]	Bipolar disorder, manic or mixed<br>Age:40±12 years<br>M: 42%<br>manic:57%<br>mixed:43%<br>Excl: history of intolerance to olanzapine or divalproex	(1) olanzapine 5-20 mg/day, n=125<br>(2) divalproex 500-2500 mg/day, n=126	[mean modal]<br>(1) 17 mg/day<br>(2) 1401 mg/day	Benzodiazepines (65%)<br>Anticholinergics for EP symptoms (13%, 10%)<br>No sig differences	3	R1, D2, W1	≥50% dec in YMRS at endpoint<br>(1) 68/125<br>(2) 52/126<br>Time to respsponse sig shorter in olanzapine group<br>Greater mean change for nonpsychotic vs psychotic patients	YMRS≤12<br>(1) 59/125<br>(2) 42/126<br>Time to remission sig shorter in olanzapine group	no data	Mean values improved in both groups. Greater improvements seen in subset with HAMD-21 ≥20 at baseline	no data
Hirschfeld et al. 2004<br>[28]	Bipolar I disorder, manic<br>Age:39±12 years<br>M: 57%<br>Excl: history of poor response to monotherapy with antimanic or antipsychotic drug	(1) risperidone 1-6 mg/day, n=134<br>(2) placebo, n=125	[mean modal]<br>4 mg/day<br>More placeo pts took max dose, and dose escalation faster	Benzodiazepines (81%, 82%)<br>Anticholinergics for EP symptoms (22%, 11%)	3	R2, D1, W1	≥50% dec in YMRS at any time<br>(1) 55/127<br>(2) 29/119<br>Greater mean change for nonpsychotic vs psychotic patients	YMRS≤12<br>(1) 48/127<br>(2) 24/119	not ill/mildy ill at endpoint:<br>(1) 68/127<br>(2) 31/119	Mean values improved in both groups	no data
Yatham et al. 2003<br>[29]	Bipolar disorder, manic or mixed<br>Age:40(19-65) years<br>M: 42%<br>manic:92%<br>mixed:8%	(1) risperidone 1-6 mg/day plus mood stabiliser, n=75<br>(2) placebo plus mood stabiliser, n=75<br>[ITT]	4 mg/day	Benzodiazepines (72%, 63%)<br>Anticholinergics for EP symptoms (16%, 8%)	3	R2, D1, W1	≥50% dec in YMRS<br>(1) 40/75<br>(2) 30/75	no data	much/v much improved at endpoint:<br>(1) 40/75<br>(2) 31/75	Mean values decreased in both groups	depressive symptoms - given antidepressants<br>(1) 1/75<br>(2) 2/75

Sachs et al.<br>2002<br>[30]	Bipolar disorder, manic or mixed<br>Age:43(18-66) years<br>M: 51%<br>manic:79%<br>mixed:21%<br>Excl: sensitivity to any trial drug, lab value outside normal range	(1) risperidone 1-6 mg/day plus mood stabiliser, n=52<br>(2) haloperidol 2-12 mg/day plus mood stabiliser, n=53<br>(3) placebo plus mood stabiliser, n=51<br>[ITT]	[mean modal]<br>(1) 4 mg/day<br>(2) 6 mg/day	Benzodiazepines (67%, 64%, 59%)<br>Anticholinergics for EP symptoms (17%, 20%, 8%)<br>sig diff tween (2) and (3) for antichol	3	R1,<br>D1,<br>W1	mean data anly	no data	much/v much improved at endpoint:<br>(1) 27/51<br>(2) 25/50<br>(3) 14/47	no data	no data
Sachs et al.<br>2004<br>[31]	Bipolar disorder, manic<br>Age:41 years<br>M: 56%<br>Excl: intolerance or lack of response to quetiapine or clozapine, rapid cycling	(1) quetiapine 200-800 mg/day plus mood stabiliser, n=90<br>(2) placebo plus mood stabiliser, n=100<br>[ITT]	[mean last week dose]<br>504 mg/day<br>No difference in use of mood stabilisers	Benzodiazepine and other sleep medication use limited and not sig diff in serum levels bet groups<br>No anticholinergics	3	R1,<br>D2,<br>W1	≥50% dec in YMRS at endpoint<br>(1) 44/81<br>(2) 29/89	YMRS≤12<br>(1) 37/81<br>(2) 23/89	much/v much improved<br>(1) 41/81<br>(2) 28/89	Total scores decreased in both groups	MADRS ≥18 with inc ≥4 from baseline on two consec assess or at endpoint<br>(1) 14/90<br>(2) 12/100
Yatham et al.<br>2004<br>[32]	Bipolar I disorder, manic<br>Age:40(18-70) years<br>M: 53%<br>Excl: intolerance or lack of response to quetiapine, lithium, divalproex or clozapine	(1) quetiapine 200-800 mg/day plus mood stabiliser, n=185<br>(2) placebo plus mood stabiliser, n=185<br>[ITT]	[mean last week dose in responders]<br>(1) 492 mg/day<br>No difference in serum levels of mood stabilisers	Benzodiazepines, no anticholinergics	3	R1,<br>D1,<br>W1	≥50% dec in YMRS at endpoint<br>(1) 103/185<br>(2) 77/185	YMRS≤12<br>(1) 90/185<br>(2) 61/185	much/v much improved<br>(1) 108/185<br>(2) 80/185	Mean baseline values low, similar reduction for both groups	MADRS ≥18 with inc ≥4 from baseline on two consec assess or at endpoint<br>(1) 19/185<br>(2) 18/185
Keck et al. 2003<br>[33]	Bipolar I disorder, manic or mixed<br>Age:38(19-64) years<br>M: 54%<br>manic:65%<br>mixed:35%<br>Excl: history of hypersensitivity to antipsychotic drugs, previous treatment with ziprasidone	(1) ziprasidone 40-80 mg/day, n=131<br>(2) placebo, n=66<br>[ITT]	[mean last week dose<br>130 mg/day	Benzodiazepines, no sig diff bet groups<br>Anticholinergics for EP symptoms	3	R2,<br>D2,<br>W1	≥50% dec in YMRS at endpoint<br>(1) 66/131<br>(2) 23/66	no data	mean changes in CGI-BP scales given, no categorical data<br>Overall: ziprasidone better than placeo	Sig greater red in mean score for ziprasidone than placebo	no data
Keck et al.<br>2003b<br>[34]	Bipolar I disorder, manic or mixed<br>Age:40±12 years<br>M: 44%<br>manic: 67%<br>mixed: 33%<br>Excl: non response to clozapine, anticipated requirement for prohibited drugs	(1) aripiprazole 30 mg/day, n=127<br>(2) placebo, n=127<br>[ITT]	[mean dose at endpoint]<br>28 mg/day	Benzodiazepines (86%, 85%)<br>Anticholinergics for EP symptoms	3	R1,<br>D1,<br>W1	≥50% dec in YMRS<br>(1) 51/127<br>(2) 24/127	no data	mean changes in CGI-BP scales given, no categorical data<br>Overall: aripiprazole better than placeo	no data	no data
Potkin et al.<br>2005<br>[35]	Bipolar I disorder, manic or mixed<br>Age: 39±12 years<br>M: 51%<br>manic: 60%<br>mixed: 40%	(1) ziprasidone 80-160 mg/day, n=139<br>(2) placebo, n=66<br>[ITT]	[mean]<br>(1) 112 mg/day<br>(2) "slightly higher"	Benzodiazepines (73%, 62%)<br>Anticholinergics for EP symptoms (25%, 6%)	3	R2,<br>D2,<br>W1	≥50% dec in MRS (SADS-CB)<br>(1) 64/139<br>(2) 19/66	no data	mean changes in CGI-BP scales given, no categorical data<br>Overall: ziprasidone better than placeo	Mean improvement greater for ziprasidone throughout study, but not sig	no data

Khanna et al.<br>2005<br>[36]	Bipolar I disorder, manic or mixed<br>Age: 35 years<br>M: 62%<br>manic: 96%<br>mixed: 4%<br>Excl: rapid cycling, antidepressant-induced mania	(1) risperidone 1-6 mg/day, n=146<br>(2) placebo, n=144	no data	Benzodiazepines (99%)<br><br>Antiparkinsonian med for EP symptoms (36%, 6%)	3	R1,<br>D1,<br>W1	≥50% dec in YMRS at endpoint<br>(1) 107/146<br>(2) 52/144<br>No sig diff between psychotic/non-psychotic and manic/mixed subgroups	no data	not ill/mildy ill at endpoint:<br>(1) 104/146<br>(2) 356/144	Mean baseline scores low, but improvement sig better in risperidone group than placebo	no data
Smulevich et al.<br>2005<br>[37]	Bipolar I disorder, manic or mixed<br>Age: 40 years<br>M: 52%<br>manic/mixed: not given<br>Excl: history poor response to antipsychotic monotherapy, rapid cycling	(1) risperidone 1-6 mg/day, n=154<br>(2) haloperidol 2-12 mg/day, n=144<br>(3) placebo, n=140	(1) 4 mg/day<br>(2) 8 mg/day	Benzodiazepines (50%)<br>Antiparkinsonian med for EPS	3	R1,<br>D1,<br>W1	≥50% dec in YMRS at 21 days<br>(1) 74/154<br>(2) 68/144<br>(3) 46/140<br><br>No sig diff in change for nonpsychotic vs psychotic patients taking risperidone	no data	mean changes in CGI-BP scales given, no categorical data<br>Overall: risperidone and haloperidol better than placeo, no sig diff between risperidone and haloperidol	Mean improvement in all groups: sig more in active groups than placebo	no data
Sachs et al.<br>2006<br>[38]	Bipolar I disorder, manic or mixed relapse, requiring hospitalisation, with YMRS≥20 at randomisation<br>Age: 39 years<br>M: 49%<br>manic: 58%<br>mixed: 42%<br>rapid cyclinng: 18%<br>Excl: history of no response to clozapine, previousaripiprazole trial	(1) aripiprazole 15-30 mg/day, n=136<br>(2) placebo, n=133	(1) 27.7 mg/day	Lorazepam	3	R1,<br>D1,<br>W1	≥50% dec in YMRS at 21 days<br>(1) 72/136<br>(2) 42/132<br><br>Sig diff between groups by 7 days, and inc to 21 days<br><br>Subgroups with rapid cycling and mixed episodes showed sig benefits for aripiprazole over placebo	no data	mean changes in CGI-BP scales sig greater for aripiprazole by day 7, no categorical data	Baseline scores low, non-sig improvement	no data
**Six to 12 weeks**											
Tohen et al.<br>2002b<br>[39]	Bipolar disorder, manic or mixed, with inadequate response to monotherapy<br>Age:41±11 years<br>M: 48%<br>manic: 52%<br>mixed: 48%	(1) olanzapine 5-20 mg/day plus mood stabiliser, n=229<br>(2) placebo plus mood stabiliser, n=115	10 mg/day	Benzodiazepines (29%, 34%)<br>Antiparkinsonian med for EPS	6	R1,<br>D1,<br>W1	≥50% dec in YMRS<br>(1) 149/220<br>(2) 51/114<br>Median time to response (days)<br>(1) 18<br>(2) 28<br>Cotherapy better than mono for non-psychotic patients	YMRS≤12<br>(1) 173/220<br>(2) 75/114<br>Median time to remission (days)<br>(1) 14<br>(2) 22	mean changes in CGI-BP scales given, no categorical data	sig improvement in mean HAMD-21 for olanzapine group<br><br>For subset with mixed episode and HAMD-21 ≥20 at baseline (n=21), sig greater reduction in group with olanzapine	As AE<br>(1) 41/225<br>(2) 20/115
Zajecka et al.<br>2002<br>Efficacy to 3 weeks, Safety to 12 weeks in responders<br>[40]	Bipolar I disorder, manic or mixed<br>Age:38±12 years<br>M: 54%<br>manic:52%<br>mixed:48%<br>Excl: previous non-responders to olanzapine or divalproex	(1) olanzapine 5-20 mg/day, n=57<br>(2) divalproex 20 mg/kg/day-20 mg/kg/day+1000mg, n=63<br>First 3 weeks as inpatient	[mean maximum]<br>(1) 15 (5-25) mg/day<br>(2) 2115 (750-3250) mg/day	No sig diff between groups for rescue meds	12	R1,<br>D2,<br>W1	Change in MRS from baseline, at 3 weeks: both groups had decrease of about 50% with no sig diff between groups. Improvements maintained to 12 weeks, with no diff between groups	no data	Mean improvement in both groups: no sig diff, maintained to 12 weeks	Mean improvement in both groups: no sig diff, maintained to 12 weeks	incomplete data, but one case of serious depression in each group

Tohen et al.<br>2003<br>[41]	Bipolar I disorder, manic or mixed<br>Age:40±13(18-86) years<br>M: 40%<br>manic:94%<br>mixed:6%	(1) olanzapine 5-20 mg/day, n=234<br>(2) haloperidol 3-15 mg/day, n=219	[median at week 6]<br>(1) 15 mg/day<br>(2) 5 mg/day<br>[median at week 12]<br>(1) 10 mg/day<br>(2) 3 mg/day	Benzodiazepines (60%, 65%)<br>Anticholinergics for EP symptoms (18%, 60%)	12	R2,<br>D1,<br>W1	≥50% dec in YMRS at 6 weeks<br>(1) 169/234<br>(2) 162/219<br>at 12 weeks<br>(1) 154/160<br>(2) 130/138	YMRS≤12 and HAM-D ≤8 at 6weeks<br>(1) 112/234<br>(2) 101/219<br>Median time to remission (days)<br>(1) 34<br>(2) 29<br>Olanzapine sig better than haloperidol for non-psychotic patients	no data	Mean baseline values low. Small mean changes not likely to be clinically relevant<br>For subgroups with mixed episode or HAMD-21 ≥20 at baseline, greater changes seen	affective episode<br>(YMRS ≥15 and HAM-D ≥15 foll remission)<br>(1) 16/122<br>(2) 15/101<br>Switch to depression<br>(HAM-D ≥15 foll ≤8 at entry<br>(1) 12/128<br>(2) 22/131
Bowden et al.<br>2005<br>[42]	Bipolar I disorder, manic<br>Age:39(18-73) years<br>M: 57%<br>Excl: intolerance to quetiapine or lithium, or lack of response to clozapine, quetiapine or lithium. Clinically sig abbnormalities in ECG or labvalues	(1) quetiapine 100-800 mg/day, n=107<br>(2) placebo, n=95<br>(3) lithium to give 0.6-1.4 mEq/L, n=98	(1) [mean last week dose in responders]<br>3 weeks: 586 mg/day<br>12 weeks: 618 mg/day<br>(2) [median serum conc]<br>3 weeks: 0.8 mEq/L<br>12 weeks: 0.8 mEq/L	Benzodiazepines (36%, 31%, 46%)<br>Anticholinergics for EP symptoms (11%, 12%, 8%)	12	R1,<br>D2,<br>W1	≥50% dec in YMRS at 21 days<br>(1) 57/107<br>(2) 26/95<br>(3) 52/98<br>at 84 days<br>(1) 77/107<br>(2) 39/95<br>(3) 74/98	YMRS≤12 at 21 days<br>(1) 50/107<br>(2) 21/95<br>(3) 48/98<br>at 84 days<br>(1) 74/107<br>(2) 32/95<br>(3) 71/98	much/v much improved at 21 days<br>(1) 68/107<br>(2) 29/95<br>(3) 63/98<br>at 84 days<br>(1) 77/107<br>(2) 35/95<br>(3) 71/98	Mean baseline values low. At 3 weeks quetiapine sig better than placebo, at 84 days, both quetiapine and lithium better than placebo	MADRS ≥18 with inc ≥4 from baseline on two consec assess or at endpoint<br>(1) 6/107<br>(2) 8/97<br>(3) 3/99<br><br>As AE:<br>(1) 6/107<br>(2) 1/97<br>(3) 1/98
McIntyre et al.<br>2005<br>[43]	Bipolar I disorder, manic<br>Age:43(18-79) years<br>M: 37%<br>Excl: rapid cycling, mixed episode, intolerance or lack of response to quetiapine or clozapine	(1) quetiapine 100-800 mg/day, n=102<br>(2) placebo, n=101<br>(3) haloperidol 2-8 mg/day, n=99	no data	Benzodiazepines (48%, 54%, 46%)<br>Anticholinergics for EP symptoms (10%, 53%, 12%)	12	R1,<br>D2,<br>W1	≥50% dec in YMRS at 21 days<br>(1) 43/101<br>(2) 35/100<br>(3) 55/98<br>at 84 days<br>(1) 62/101<br>(2) 39/100<br>(3) 69/98<br>Greater mean change for nonpsychotic vs psychotic patients	YMRS≤12 at 21 days<br>(1) 28/101<br>(2) 24/100<br>(3) 36/98<br>at 84 days<br>(1) 62/101<br>(2) 38/100<br>(3) 62/98	much/v much improved at 21 days<br>(1) 44/101<br>(2) 35/100<br>(3) 52/98<br>at 84 days<br>(1) 51/101<br>(2) 30/100<br>(3) 59/98	Mean baseline values low. At 3 weeks quetiapine and haloperidol sig better than placebo, at 84 days, only quetiapine better than placebo	MADRS ≥18 with inc ≥4 from baseline on two consec assess or at endpoint<br>(1) 3/102<br>(2) 9/101<br>(3) 8/99<br><br>As AE:<br>(1) 2/102<br>(2) 4/101<br>(3) 1/99
Vieta et al. 2005<br>[44]	Bipolar I disorder, manic or mixed<br>Age:42 (0.6) years<br>M: 38%<br>manic:89%<br>mixed:11%<br>Excl: rapid cycling, considered unresponsive to antipsychotics	(1) aripiprazole 15-30 mg/day, n=174<br>(2) haloperidol 10-15 mg/day, n=170<br>[ITT]	no data	Benzodiazepines<br><br>No anticholinergics for sympt or prophylact treatment of EP sympt	12	R1,<br>D1,<br>W1	≥50% dec in YMRS<br>at 3 weeks<br>(1) 88/174<br>(2) 72/170<br>at 12 weeks<br>(1) 86/174<br>(2) 48/170<br>≥50% dec in MADRS<br>at 3 weeks<br>(1) 80/174<br>(2) 63/170<br>at 12 weeks<br>(1) 80/174<br>(2) 56/170	YMRS<12<br>at 3 weeks<br>(1) 61/174<br>(2) 53/170<br>at 12 weeks<br>(1) 87/174<br>(2) 46/170	mean changes in CGI-BP scales given, no categorical data	≥50% dec in MADRS<br>at 3 weeks<br>(1) 89/174<br>(2) 63/170<br>at 12 weeks<br>(1) 89/174<br>(2) 56/170<br><br>Sig diff in mean scores up to week 6, then ns diff	CGI-BP depression scale dec ≥2 points<br>(1) 19/173<br>(2) 29/164<br><br>AsAE:<br>(1) 20/175<br>(2) 24/169

Longer than 12 weeks

Tohen et al. 2003b<br>See Tohen 2002, for analysis to 3 weeks<br>[45]	Bipolar disorder, manic or mixed<br>Age:40±12 years<br>M: 42%<br>manic:57%<br>mixed:43%<br>Excl: history of intolerance to olanzapine or divalproex	(1) olanzapine 5-20 mg/day, n=125<br>(2) divalproex 500-2500 mg/day, n=126	(1) 16 mg/day<br>(2) 2585 mg/day	Benzodiazepine use limited<br>Anticholinergics to treat EP symptoms	47	R1,<br>D2,<br>W1	Mean YMRS scores sig better for olanzapine than divalproex from wk 2 to 15, then no sig diff wk 30 to 47<br><br>Mean HAM-D scores non sig better for olanzapine than divalproex	YMRS≤12<br>(1) 71/125<br>(2) 57/126<br>Median time to remission (days)<br>(1) 14<br>(2) 62<br>YMRS≤12 and MADRS≤8 at endpoint<br>(1) 39/125<br>(2) 39/126	no sig diff between groups	no data	symp relapse into affective episode (YMRS≥15 or HAM-D ≥15 foll remission)<br>(1) 14/33<br>(2) 13/23<br><br>As AE:<br>(1) 43/125<br>(2) 38/126
Tohen et al. 2004<br>See Tohen 2002b for parent study<br>[46]	Bipolar I disorder with remission of manic episode after treatment with olanzapine plus lithium or valproate<br>Age: 41 (19-69) years<br>M:48%<br>manic:50%<br>mixed:50%<br>Excl: history of intolerance to olanzapine	additional<br>(1) olanzapine 5-20 mg/day plus lithium or valproate, n=51<br>(2) placebo plus lithium or valproate, n=48	9 mg/day<br>lithium or valproate doses very similar between groups	Benzodiazepine use limited (29%, 20%)<br>Anticholinergics to treat EP symptoms (15%, 10%)	78	R2,<br>D2,<br>W1	n/a	n/a	no data	no data	Sympt relapse into affective episode (YMRS +/or HAM-D≥15) without symptoms initially<br>(1) 11/30<br>(2) 21/38<br>Median time to relapse (days)<br>(1) 163<br>(2) 42<br>Depression alone:<br>(1) 7/30 (163 days)<br>(2) 15/38 (55 days)<br>Mania alone:<br>(1) 6/30 (172 days)<br>(2) 11/38 (59 days)
Tohen et al. 2005<br>[47]	Bipolar disorder with remission of manic/mixed episode after open-label treatment with olanzapine plus lithium for 6 weeks<br>Age: 42 (13) years<br>M:47%<br>manic:93%<br>psychotic:26%<br>Excl: history of intolerance or lack of response to lithium or olanzapine	(1) olanzpine 5-20 mg/day, n=217<br>(2) lithium to give 0.6-1.4 mEq/L, n=214<br>Patients started on open-label doses of drugs, then 4 week d-b taper to monotherapy	(1) 12 mg/day<br>(2) 1103 mg/day	Benzodiazepine use limited (36%, 52%)<br>Anticholinergics to treat EP symptoms (7%, 8%)	52	R2,<br>D2,<br>W1	n/a	n/a	no data	no data	Sympt recurrence of any mood episode (YMRS +/or HAM-D≥15)<br>(1) 65/217<br>(2) 83/214<br>Mania:<br>(1) 30/217<br>(2) 50/214<br>Depression:<br>(1) 34/217<br>(2) 23/214<br>Time to recurrence not sig diff between groups

Tohen et al. 2006 [48]	Bipolar I disorder with remission of manic/mixed episode after open-label treatment with olanzapine for 6-12 weeks<br>Age: 40 (12) years<br>M:39%<br>manic:67%<br>mixed:33%<br>psychotic:18%<br>Excl: intolerance to minimum dose of olanzapine in open-lael phase	(1) olanzapine 5-20 mg/day, n=225<br>(2) placebo, n=136	12.5 mg/day	Benzodiazepine use limited (27%, 36%)<br>Anticholinergics to treat EP symptoms (4%, 4%)	48	R1, D1, W1	n/a	n/a	n/a	no data	Sympt recurrence of any mood episode (YMRS +/or HAM-D≥15)<br>(1) 105/225<br>(2) 109/136<br>Mania:<br>(1) 27/225<br>(2) 44/136<br>Depression:<br>(1) 68/225<br>(2) 53/136<br>Mixed:<br>(1) 10/225<br>(2) 12/136<br>Time to any relapse:<br>(1) 174 days<br>(2) 22 days<br><br>Olanzapine better for pts with/w'out rapid cycling and with/w'out psychotic features, and manic/mixed index episode
Keck et al. 2006 [49]	Bipolar I disorder with recent stabilisation of manic/mixed episode after treatment aripiprazole (YMRS<10, MADRS≤13 for ≥6 weeks)<br>Age: 40±1 years<br>M:33%<br>manic:70%<br>mixed:30%<br>Excl: history of cognitive disorder, schizophrenia/ schizoaffective disorder,psychoactive or substance use disorder, unresponsive to clozapine, intolerence to aripiprazole	(1) aripiprazole 15-30 mg/day, n=78<br>(2) placebo, n=83	mean 25mg/day	Lorazepam (39%, 46%)<br>Anticholinergics for EPS (39%, 31%)<br>Analgesic/antipyretic (34%, 36%)	26	R1, D1, W1	n/a	n/a	n/a	no data	Relapse (withdrawal due to LoE)<br>(1) 19/77<br>(2) 36/83<br>Manic relapse:<br>(1) 6/77<br>(2) 19/83<br>Depressive relapse:<br>(1) 9/77<br>(2) 11/83<br>Mixed/unknown:<br>(1) 4/77<br>(2) 6/83<br><br>Time to relapse sig longer for (1) than (2)

QS = quality score; R = randomised; D = double blind; W = withdrawals or drop outs; CGI = clinical global impression; TE = treatment emergent; EP = extrapyramidal; EPRS = extrapyramidal rating scale; AIMS = abnormal involuntary movement scale; SAS = Simpson-Angus rating scale; BARS = Barnes akathisia rating scale

Discontinuations				Adverse events				Scale-defined adverse events	
**All cause**	**LoE**	**AE**	**T-E depression**	**All cause**	**Severe**	**Serious**	**T-R**	**EPS**	**Weight gain ≥7%**
**no data**	**no data**	**no data**	**no data**	**no data**	**no data**	**no data**	**no data**	**SAS**<br>**Mean score in lithium group lower than in risperidone or halooperidol groups**	**no data**
(1) 27/70<br>(2) 45/69	(1) 20/70<br>(2) 33/69	(1) 0/70<br>(2) 2/69	no data	no data	no data	no data	no data	SAS, BARS, AIMS<br>Infrequent<br>No sig diff	no data
(1) 1/15<br>(2) 3/15	no data	(1) 1/15<br>(2) 1/15	no data	no data	no data	no data	no data	SAS , BARS<br>No sig diff in SAS between groups<br>Akathisia<br>(1) 0/15<br>(2) 0/15	no data
(1) 21/55<br>(2) 35/60	(1) 15/55<br>(2) 23/60	(1) 2/55<br>(2) 1/60	no data	no data	no data	no data	no data	SAS, BARS<br>Infrequent<br>No sig diff	no data
(1) 39/125<br>(2) 45/126	(1) 11/125<br>(2) 12/126	(1) 12/125<br>(2) 9/126	no data	no data	no data	no data	no data	SAS, BARS, AIMS<br>No sig diff	no data
(1) 59/134<br>(2) 73/125	(1) 19/134<br>(2) 45/125	(1) 10/134<br>(2) 7/125	no data	no data	no data	no data	no data	EPRS<br>Small but sig inc Risp vs Placebo overall but not indiv subscales	no data
(1) 12/75<br>(2) 14/75<br>Disc early and entered open<br>(1) 15/75<br>(2) 25/75	(1) 2/75<br>(2) 1/75	(1) 1/75<br>(2) 3/75	no data	(1) 43/75<br>(2) 38/75	no data	no data	no data	EPRS<br>No sig diff in means	no data

(1) 18/52<br>(2) 28/53<br>(3) 25/51	(1) 3/52<br>(2) 3/53<br>(3) 5/51	(1) 2/52<br>(2) 1/53<br>(3) 2/51	no data	(1) 42/52<br>(2) 49/53<br>(3) 43/51	no data	no data	no data	EPRS<br>Significantly greater increase with haloperidol than placebo.	no data
(1) 34/90<br>(2) 51/100	(1) 7/90<br>(2) 14/100	(1) 5/90<br>(2) 6/100	no data	no data	no data	no data	no data	SAS, BARS<br>Low levels<br>No sig diff	(1) 4/90<br>(2) 1/100
(1) 52/197<br>(2) 71/205	(1) 22/197<br>(2) 30/205	(1) 6/197<br>(2) 8/205	no data	no data	no data	no data	no data	SAS, BARS<br>Small changes<br>No sig diff	no data
(1) 65/140<br>(2) 39/70	(1) 27/140<br>(2) 25/70	(1) 9/140<br>(2) 3/70	no data	(1) 126/140<br>(2) 54/70	no data	no T-R	(1) 99/140<br>(2) 38/70	SAS, BARS, AIMS<br>Infreq<br>No sig diff	no data
(1) 76/130<br>(2) 104/132<br>Entered open-label treatment<br>(1) 17/130<br>(2) 37/132	(1) 13/130<br>(2) 16/132<br>but see open label treatment	(1) 14/130<br>(2) 13/132	no data	no data	no data	(1) 4/130<br>(2) 4/132	no data	SAS, BARS small but sig inc in (1)<br>AIMS no sig diff	(1) 2/127<br>(2) 0/127
(1) 55/140<br>(2) 30/66	(1) 18/140<br>(2) 19/66	(1) 9/140<br>(2) 1/66<br>T-R<br>(1) 8/139<br>(2) 1/66	no data	(1) 109/139<br>(2) 44/66	(1) 4/139<br>(2) 1/66	no data	(1) 90/139<br>(2) 27/66	SAS, BARS, AIMS<br>No sig diff	(1) 6/125<br>(2) 2/59

(1) 16/146<br>(2) 42/144	(1) 7/146<br>(2) 21/144	(1) 5/146<br>(2) 3/144	no data	no data	most mild to moderate	similar in both groups	no data	ESRS<br>Small, insig changes	no data
(1) 17/154<br>(2) 14/144<br>(3) 21/140	(1) 5/154<br>(2) 2/144<br>(3) 9/140	(1) 6/154<br>(2) 4/144<br>(3) 7/140	no data	no data	most mild to moderate	no data	no data	ESRS<br>Sig diff from placebo for haloperidol, not risperidone	no data
(1) 61/136<br>(2) 63/133	(1) 12/136<br>(2) 28/133	(1) 12/136<br>(2) 10/133	no data	no data	Somnolence and gi events with aripiprazole generally mild to moderate and transient	(1) 12/136<br>(2) 10/133	no data	SAS, AIMS Changes small and not sig diff between groups<br>BARS Changes small, with sig diff at endpoint, but no sig diff between number of patients with score ≥2 (clinical sig)	(1) 1/136<br>(2) 5/133
(1) 69/229<br>(2) 33/115	(1) 7/229<br>(2) 14/115	(1) 25/229<br>(2) 2/115	(1) 0/229<br>(2) 2/115	no data	no data	no data	no data	SAS, BARS, AIMS<br>No sig diff	no data
(1) 38/57<br>(2) 45/63	(1)11/57<br>(2) 14/63	(1) 5/57<br>(2) 7/63	no data	sig less with olanzapine than divalproex	no data	(1) 2/57<br>(2) 5/63<br><br>T-R<br>(1) 1/57<br>(2) 1/63	no data	SAS, BARS<br>No sig diff	no data

(1) 94/234<br>(2) 103/219	(1) 35/234<br>(2) 33/219	(1) 19/234<br>(2) 25/219	no data	no data	no data	no data	no data	SAS, AIMS, BARS<br>EPS sig worse with haloperidol<br>Parkinsonism (1) 24/187 (2) 92/169<br>Akathisia (1) 20/203 (2) 78/193<br>Dyskinesia (1) 7/223 (2) 19/208	(1) 91/229<br>(2) 34/211
(1) 35/107<br>(2) 62/97<br>(3) 31/98	(1) 16/107<br>(2) 38/97<br>(3) 12/98	(1) 7/107<br>(2) 4/97<br>(3) 6/98	no data	no data	most mild to moderate	no data	no data	SAS, BARS<br>EPS rel events<br>(1) 14/107<br>(2) 9/97<br>(3) no data<br>Overall no sig diff between quetiapine and placebo. More tremor in lithium group	no data
(1) 47/102<br>(2) 59/101<br>(3) 45/99	(1) 27/102<br>(2) 43/101<br>(3) 25/99	(1) 5/102<br>(2) 6/101<br>(3) 10/99	(1) 1/102<br>(2) 3/101<br>(3) 0/99	no data	no data	no data	no data	SAS, BARS<br>No sig diff bet (1) and (2), but (3) sig worse	(1) 13/102<br>(2) 4/101<br>(3) 5/99
at 3 weeks<br>(1) 41/175<br>(2) 77/172<br>at 12 weeks<br>(1) 86/175<br>(2) 122/172<br><br>Time to disc greater for aripiprazole than haloperidol	(1) 30/175<br>(2) 10/172	(1) 32/175<br>(2) 84/172<br>T-R<br>(1) 0/175<br>(2) 1/172	no data	no data	no data	(1) 6/175<br>(2) 12/172	no data	SAS, BARS, AIMS show minimal change for aripiprazole, sig worsening for haloperidol	no data

(1) 106/125<br>(2) 106/127	(1) 24/125<br>(2) 28/126	(1) 31/125<br>(2) 25/126	no data	no data	no data	no data	no data	SAS, BARS, AIMS<br>No sig diff	(1) 29/123<br>(2) 22/123
(1) 35/51<br>(2) 43/48	(1) 13/51<br>(2) 17/48	(1) 5/51<br>(2) 8/48	no data	no data	no data	no data	no data	SAS, BARS<br>No sig diff<br>(Akathisia)<br>(1) 4/51<br>(2) 3/48<br>(Parkinsonism)<br>(1) 3/51<br>(2) 4/48<br>(Dyskinesia)<br>(1) 0/51<br>(2) 2/48	(1) 14/51<br>(2) 3/48
(1) 116/217<br>(2) 144/214	(1) 31/217<br>(2) 34/214	(1) 41/217<br>(2) 55/214	no data	no data	no data	no data	no data	SAS, BARS, AIMS<br>Changes small and not sig diff between groups<br>(Akathisia)<br>(1) 0/189<br>(2) 4/197<br>(Parkinsonism)<br>(1) 6/177<br>(2) 5/176<br>(Dyskinesia)<br>(1) 3/206<br>(2) 2/209	(1) 64/217<br>(2) 21/214

(1) 72/225<br>(2) 18/136<br><br>19/225 olanzapine pts lost to foll up, vs 5/136 placebo	(1) 4/225<br>(2) 2/136	(1) 17/225<br>(2) 0/136	no data	no data	no data	(1) 7/225<br>(2) 13/136<br><br>most due to worsening of illness	no data	SAS, BARS, AIMS<br>Few events, not sig diff between groups<br>(Akathisia)<br>(1) 9/194<br>(2) 1/119<br>(Parkinsonism)<br>(1) 5/206<br>(2) 0/118<br>(Dyskinesia)<br>(1) 0/216<br>(2) 1/113	(1) 36/225<br>(2) 3/136
(1) 39/78<br>(2) 55/83	(1) 19/78<br>(2) 36/83	(1) 5/78<br>(2) 1/83	(1) <3%<br>(2) 6/83	(1) 57/77<br>(2) 58/83	no data	(1) 6/77<br>(2) 11/83	no data	SAS, AIMS, BARS changes minimal<br>SAS≥15 (moderate severity):<br>(1) 7/76<br>(2) 6/81<br>BARS≥3 (moderate to severe):<br>(1) 4/76<br>(2) 2/81<br><br>EPS more frequent with aripiprazole than placebo<br>(Akathisia)<br>(1) 5/77<br>(2) 1/83<br>(Tremor)<br>(1) 7/77<br>(2) 1/83<br>(Hypertonia)<br>(1) 3/77<br>(2) 1/83	(1) 7/56<br>(2) 0/60
