# Supplementary material for: Atypical antipsychotics in bipolar disorder: systematic review of randomised trials
Source: BMC Psychiatry. 2007 Aug 16;7:40. doi: 10.1186/1471-244X-7-40 (PMC2020469; doi:10.1186/1471-244X-7-40)
Supplement: Additional file 3 — Individual adverse events in all trials. A listing of the individual adverse events reported in the trials. [file 1471-244X-7-40-S3.pdf]

Additional file 3: Individual adverse events

Study	Drug comparison	Method of collection	Category reported	Somnolence	Weight gain	Increased appetite	Headache	Dry mouth	Nervousness	Asthenia	Anxiety	Depression	Insomnia	Diarrhoea	Nausea	Vomiting
**Mania/mixed: less than 6 weeks**																
Segal et al, 1998	risperidone v lithium v haloperidol	Documented	not reported													
Tohen et al. 1999	olanzapine v placebo	no information	in ≥10% of group or stat sig between-group diff	(1) 23/70<br>(2) 12/69	(1) 8/70<br>(1) 1/69		(1) 12/70<br>(2) 11/69	(1) 18/70<br>(2) 6/69	(1) 6/70<br>(2) 9/69	(1) 13/70<br>(2) 16/69	(1) 10/70<br>(2) 7/69	(1) 9/70<br>(2) 8/69				
Berk et al, 1999	olanzapine v lithium	Noted	not reported													
Tohen et al. 2000	olanzapine v placebo	Assessed	in ≥10% of group or stat sig between-group diff	(1) 21/55<br>(2) 5/60	sig larger mean weight gain for olanzapine		(1) 10/55<br>(2) 13/60	(1)9/55<br>(2) 3/55	(1) 5/55<br>(2) 12/60	(1) 6/55<br>(2) 3/60	(1) 2/55<br>(2) 9/60					
Tohen et al. 2002	olanzapine v divalproex	Monitored	in ≥10% of group or stat sig between-group diff	(1) 49/125<br>(2) 26/126	(1) 15/125<br>(2) 10/126	(1) 15/125<br>(2) 3/126	(1) 28/125<br>(2) 29/126	(1) 42/125<br>(2) 8/126	(1) 13/125<br>(2) 21/126	(1) 20/125<br>(2) 17/126			sleep disorder<br>(1) 7/125<br>(2) 1/126	(1) 8/125<br>(2) 17/126	(1) 13/125<br>(2) 36/126	(1) 10/125<br>(2) 18/126
Hirschfeld et al. 2004	risperidone v placebo	General and directed questioning	in ≥10% of group	(1) 38/134<br>(2) 9/125			(1) 19/134<br>(2) 19/125								(1) 15/134<br>(2) 3/125	
Yatham et al. 2003	risperidone+MS v placebo+MS	no information	most frequent		sig larger mean weight gain for risperidone		(1) 9%<br>(2) 9%					given antidepressant<br>(1) 1/75<br>(2) 2/75	(1) 4%<br>(2) 8%		(1) 5%<br>(2) 3%	
Sachs et al. 2002	risperidone+MS v haloperidol+MS v placebo+MS	Obtained	in ≥10% of group	(1) 13/52<br>(2) 16/53<br>(3) 6/51	sig larger mean weight gain for risperidone		(1) 11/52<br>(2) 8/53<br>(3) 12/51									
Sachs et al. 2004	quetiapine+MS v placebo+MS	Examination and questioning every study day	in ≥10% of group	(1) 36/90<br>(2) 10/100			(1) 24/90<br>(2) 21/100	(1) 17/90<br>(2) 4/100		(1) 10/90<br>(2) 3/100						
Yatham et al. 2004	quetiapine+MS v placebo+MS	Examinatioin, patient reports, medical records	in ≥5% of group (1) and x2 incidence in group (2)	(1) 66/196<br>(2) 19/203	(1) 12/196<br>(2) 5/203			(1) 38/196<br>(2) 6/203		(1) 19/196<br>(2) 8/203						
Keck et al. 2003	ziprasidone v placebo	Observed or reported	in ≥10% of group	(1) 52/140<br>(2) 9/70	no sig diff		(1) 30/140<br>(2) 13/70						(1) 11/140<br>(2) 7/70		(1) 16/140<br>(2) 7/70	
Keck et al. 2003b	aripiprazole v placebo	Obtained	in ≥10% of group	(1) 26/127<br>(2) 6/127			(1) 46/127<br>(2) 40/127				(1) 23/127<br>(2) 13/127		(1) 19/127<br>(2) 11/127	(1) 15/127<br>(2) 11/127	(1) 29/127<br>(2) 13/127	(1) 20/127<br>(2) 6/127
Potkin et al. 2005	ziprasidone v placebo	Observed or volunteered	T-R in ≥5% of group	(1) 31/139<br>(2) 4/66			(1) 17/139<br>(2) 5/66			(1) 7/139<br>(2) 1/66					(1) 9/139<br>(2) 1/66	

Khanna et al. 2005	risperidone v placebo	no information	in ≥10% of group		no sig diff								(1) 9/146<br>(2) 14/144			
Smulevich et al. 2005	risperidone v haloperidol v placebo	Monitored and assessed	in ≥10% of active treatment groups	(1) 7/154<br>(2) 5/144<br>(3) 2/140												
Sachs et al. 2006	aripiprazole v placebo	Obtained and measured	in ≥10% of group	(1) 27/136<br>(2) 14/133			(1) 34/136<br>(2) 33/133				(1) 14/136<br>(2) 11/133			(1) 9/136<br>(2) 13/133	(1) 29/136<br>(2) 21/133	(1) 15/136<br>(2) 10/133
**Mania/mixed: 6-12 weeks**																
Tohen et al. 2002b	olanzapine+MS v placebo+MS	no information	in ≥10% of group or stat sig between-group diff	(1) 118/229<br>(2) 31/115	(1) 60/229<br>(2) 8/115	(1) 54/229<br>(2) 9/115	(1) 36/229<br>(2) 21/115	(1) 73/229<br>(2) 9/115	(1) 24/229<br>(2) 17/115	(1) 42/229<br>(2) 15/115		(1) 41/229<br>(2) 20/115		(1) 27/229<br>(2) 17/115		
Zajecka et al. 2002	olanzapine v divalproex	spontaneous reports and monitoring	stat sig diff between groups	(1) 27/57<br>(2) 18/63	(1) 14/57<br>(2) 6/63											
Tohen et al. 2003	olanzapine v haloperidol	non-directed, open-ended questioning, spontaneous report, clinical observation	in ≥10% of group	(1) 35/234<br>(2) 19/219	(1) 32/234<br>(2) 9/219											
Bowden et al. 2005	quetiapine v placebo v lithium	questioning and examination	in ≥5% of group	(1) 21/107<br>(2) 3/97<br>(3) 9/98	(1) 16/107<br>(2) 1/97<br>(3) 6/98		(1) 8/107<br>(2) 4/97<br>(3) 12/98	(1) 26/107<br>(2) 2/97<br>(3) 6/98		(1) 7/107<br>(2) 1/97<br>(3) 4/98		(1) 6/107<br>(2) 1/97<br>(2) 1/98	(1) 10/107<br>(2) 20/97<br>(3) 16/98	(1) 5/107<br>(2) 4/97<br>(3) 5/98	(1) 1/107<br>(2) 2/97<br>(3) 6/98	(1) 1/107<br>(2) 2/97<br>(3) 6/98
McIntyre et al. 2005	quetiapine v placebo v haloperidol	open-ended questioning, spontaneous report, clinical observation	in ≥5% of active treatment groups	(1) 13/102<br>(2) 5/101<br>(3) 9/99			(1) 5/102<br>(2) 4/101<br>(3) 8/99	(1) 7/102<br>(2) 4/101<br>(3) 4/99				(1) 2/102<br>(2) 4/101<br>(3) 1/99	(1) 20/102<br>(2) 20/101<br>(3) 14/99			

Vieta et al. 2005	aripiprazole v haloperidol	reports gathered, measured	in ≥10% of group		small mean changes, not sig diff		(1) 19/174<br>(2) 20/172						(1) 24/175<br>(2) 12/172			
**Mania/mixed: more than 12 weeks**																
Tohen et al. 2003b	olanazpine v divalproex	Assessed	in ≥10% of group or stat sig between-group diff	(1) 58/125<br>(2) 31/126	(1) 31/125<br>(2) 15/126	(1) 17/125<br>(2) 7/126	(1) 33/125<br>(2) 34/126	(1) 43/125<br>(2) 9/126	(1) 15/125<br>(2) 28/126	(1) 29/125<br>(2) 23/126	(1) 16/125<br>(2) 22/126	(1) 43/125<br>(3) 38/126	(1) 10/125<br>(2) 20/126	(1) 13/125<br>(2) 24/126	(1) 20/125<br>(2) 40/126	(1) 17/125<br>(2) 23/126
Tohen et al. 2004	olanzapine+MS v placebo+MS	no information	in ≥10% of group	(1) 10/51<br>(2) 4/48	(1) 10/51<br>(2) 3/48					(1) 5/51<br>(2) 6/48	(1) 7/51<br>(2) 7/48	(1) 19/51<br>(2) 14/48	(1) 2/51<br>(2) 13/48	(1) 5/51<br>(2) 8/48		
Tohen et al. 2005	olanzapine v lithium	Reported	in ≥5% of either group	[hypersomnia]<br>(1) 6/217<br>(2) 0/214	(1) 14/217<br>(2) 10/214		(1) 9/217<br>(2) 11/214				(1) 12/217<br>(2) 10/214	(1) 45/217<br>(2) 25/214	(1) 17/217<br>(2) 48/214		(1) 1/217<br>(2) 8/214	
Tohen et al. 2006	olanzapine v placebo	Spontaneous report	in ≥10% of group(most in open label phase)	(1) 6/225<br>(2) 2/136	(1) 18/225<br>(2) 2/136	(1) 4/225<br>(2) 0/136		(1) 4/225<br>(2) 1/136					(1) 5/225<br>(2) 19/136			
Keck et al. 2006	aripiprazole v placebo	Asked about adverse events and observed	≥5% in either group	(1) 4/77<br>(2) 6/83			(1) 6/77<br>(2) 13/83		(1) 8/77<br>(2) 5/83	(1) 6/77<br>(2) 7/83	(1) 13/77<br>(2) 12/83	(1) 9/77<br>(2) 12/83	(1) 12/77<br>(2) 16/83		(1) 7/77<br>(2) 4/83	
**Depression: 8-12 weeks**																
Tohen et al. 2003d	olanzapine v olanzapine+fluoxetine v placebo	Recorded, assessed	in ≥10% of group	(1) 104/351<br>(2) 18/82<br>(3) 47/355		(1) 50/351<br>(2) 11/82<br>(3) 19/355	(1) 46/351<br>(2) 12/82<br>(3) 70/355	(1) 41/351<br>(2) 12/82<br>(3) 70/355	(1) 39/351<br>(2) 8/82<br>(3) 30/355	(1) 36/351<br>(2) 11/82<br>(3) 12/355			(1) 31/351<br>(2) 8/82<br>(3) 57/355	(1) 24/351<br>(2) 16/82<br>(3) 25/355	(1) 16/351<br>(2) 10/82<br>(3) 33/355	
Shelton et al. 2004	risperidone v parox v risperidone+paroxetine	not specified	all {not all shown here}	(1) 5/10<br>(2) 2/10<br>(3) 2/10	(1) 1/10<br>(2) 1/10<br>(3) 4/10	(1) 2/10<br>(2) 2/10<br>(3) 2/10	(1) 1/10<br>(2) 1/10<br>(3) 0/10	(1) 1/10<br>(2) 3/10<br>(3) 1/10			(1) 1/10<br>(2) 0/10<br>(3) 0/10		(1) 0/10<br>(2) 2/10<br>(3) 1/10	(1) 2/10<br>(3) 3/10<br>(3) 1/10	(1) 0/10<br>(2) 2/10<br>(3) 0/10	

Calabrese et al. 2005	quetiapine600 v quetiapine300 v placebo	Assessed	in ≥10% of group	(1) 44/180<br>(2) 49/179<br>(3) 15/180			(1) 18/180<br>(2) 22/179<br>(3) 36/180	(1) 73/180<br>(2) 79/179<br>(3) 14/180		(1) 6/180<br>(2) 14/179<br>(3) 23/180
Brown et al. 2006	olanzapine+fluoxetine v lamotrigine	nonprobing inquiry	In >5% of either group	(1) 38/205<br>(2) 17/204	(1) 29/205<br>(2) 4/204	(1) 36/205<br>(2) 17/204	(1) 24/205<br>(2) 19/204	(1) 32/205<br>(2) 12/204	(1) 9/205<br>(2) 18/204	(1) 16/205<br>(2) 16/204
Thase et al. 2006	quetiapine600 v quetiapine300 v placebo	Evaluated	in ≥10% of any group	(1) 50/168<br>(2) 51/171<br>(3) 8/167			(1) 14/168<br>(2) 15/171<br>(3) 28/167	(1) 79/168<br>(2) 73/171<br>(3) 30/167		(1) 18/168<br>(2) 13/171<br>(3) 22/167

EP = extrapyramidal;

Dyspepsia	Constipation	Dizziness	Agitation	Fatigue	Pain	Hostility	Personality disorder	Pharyngitis	Tongue oedema	Postural hypotension	Accidental injury	Prolactin	Glucose	Lipids	Extrapyramidal symptom related
	(1) 8/70<br>(2) 2/69	(1) 16/70<br>(2) 4/69	(1) 13/70<br>(2) 16/69		(1) 8/70<br>(2) 3/69	(1) 6/70<br>(2) 8/69	(1) 5/70<br>(2) 8/69								
(2) 7/55<br>(2) 3/60	(1) 6/55<br>(2) 5/60	(1) 7/55<br>(2) 4/60	(1) 5/55<br>(2) 15/60			(1) 1/55<br>(2) 6/60	(1) 1/55<br>(2) 7/60								
(1) 18/125<br>(2) 14/126	(1) 18/125<br>(2) 15/126	(1) 20/125<br>(2) 15/126	(1) 14/125<br>(2) 14/126		(1) 17/125<br>(2) 18/126				(1) 6/125<br>(2) 0/126						Tremor (1) 12/125 (2) 4/126<br>Neck rigidity (1) 9/125 (2) 2/126<br>Speech disorder (1) 10/125 (2) 1/126
(1) 15/134<br>(2) 8/125		(1) 15.134<br>(2) 11/125										sig inc in mean for risperidone group	no sig diff		Hyperkinesia (1) 21/134 (2) 6/125
															EP rel (1) 16/75 (2) 6/75
(1) 9/52<br>(2) 9/53<br>(3) 9/51	(1) 2/52<br>(2) 6/53<br>(3) 2/51	(1) 7/52<br>(2) 4/53<br>(3) 1/51													EP disorder (1) 7/52 (2) 15/53<br>(3) 2/51<br>Tremor (1) 2/52 (2) 6/53 (3) 2/51
		(1) 9/90<br>(2) 6/100								(1) 10/90<br>(2) 3/100			no sig diff		
								(1) 11/196<br>(2) 5/203		(1) 13/196<br>(2) 3/203					E-P rel (1) 42/196 (2) 39/203<br>[Akathisia (1) 7/196 (2) 10/203]
(1) 14/140<br>(2) 7/70		(1) 38/140<br>(2) 7/70													Akathisia (1) 15/140 (2) 4/70<br>Hypertonia (1) 16/140 (2) 2/70
(1) 28/127<br>(2) 13/127	(1) 17/127<br>(2) 7/127	Lightheadedness<br>(1) 18/127<br>(2) 10/127	(1) 25/127<br>(2) 24/127								(1) 15/127<br>(2) 3/127	above ULN:<br>(1) 14/127<br>(2) 22/127<br>both gps dec	no sig diff	cholesterol - no sig diff	Akathisia (1) 14/127 (2) 3/127<br>Tremor (1) 8/127 (2) 4/127
		(1) 14/139<br>(2)1/66			abbdominal<br>(1) 2/139<br>(2) 5/66									elevated triglycerides<br>(1) 8/139<br>(2) 7/66	EP syndrome (1) 15/139 (2) 1/66<br>Akathisia (1) 13/139 (2) 3/66<br>Tremor (1) 11/139 (2) 1/66

							inc in risperidone group			EP disorder (1) 51/146 (2) 9/145<br>Tremor (1) 15/146 (2) 1/145<br>Dystonia (1) 7/146 (2) 0/145<br>Hyperkinesia (1) 1/146 (2) 0/145
							inc in both active groups, more in risperidone			EP disorder (1) 26/154 (2) 58/144 (3) 12/140<br>Hyperkinesia (1) 14/154 (2) 22/144 (3) 4/140<br>Tremor (1) 10/154 (2) 16/144 (3) 8/140<br>Hypertonia (1) 6/154 (2) 13/144 (3) 0/140
(1) 21/136<br>(2) 9/133	(1) 16/136<br>(2) 7/133		(1) 20/136<br>(2) 19/133	extremity<br>(1) 14/136<br>(2) 7/133			dec in both groups, stat greater with aripiprazole<br>≥ULN<br>(1) 5/136<br>(2) 15/133	no clinically sig diff	no clinically sig diff	Akathisia (1) 24/136 (2) 6/133
		(1) 31/229<br>(2) 8/115					>ULN<br>(1) 43/229<br>(2) 5/115	no sig diff		Tremor (1) 53/229 (2) 15/115<br>Speech disorder (1) 15/229 (2) 1/115
					Oedema<br>(1) 8/57<br>(2) 0/63			no sig diff overall, but one olanzapine patient died from diabetic ketoacidosis	cholesterol- sig increase in mean total and LDL for olanzapine group	Speech disorder (1) 4/57 (2) 0/63
								<200 to ≥200 mg/dl<br>(1) 3/209<br>(2) 4/194	cholesterol <200 to ≥240 mg/dl<br>(1) 9/134<br>(2) 8/133	Akathisia (1) 15/234 (2) 65/219<br>Tremor (1) 13/234 (2) 34/219<br>Hypertonia (1) 12/234 (2) 39/219<br>EP syndrome (1) 5/234 (2) 52/219<br>Dystonia (1) 3/234 (2) 15/219<br>Dyskinesia (1) 1/234 (2) 7/219<br>Hypokinesia [sic] (1) 1/234 (2) 8/219<br>Tardive dyskinesia (1) 0/234 (2) 5/219
		(1) 13/107<br>(2) 2/97<br>(3) 7/98					Mean levels high at baseline<br>Shift to >ULN:<br>(1) 6/53<br>(2) 6/47<br>(3) 7/55	no clinically important differences		Tremor (1) 6/107 (2) 4/97 (3) 18/98<br>Akathisia (1) 1/107 (2) 6/97 (2) 3/98
			(1) 8/102<br>(2) 9/101<br>(3) 8/99			(1) 6/102<br>(2) 1/101<br>(3) 2/99	Mean levels high at baseline, dec in all groups, but more in quetiapine group			EPS-rel (1) 13/102 (2) 16/101 (3) 59/99<br>Tremor (1) 8/102 (2) 6/101 (3) 30/99<br>Akathisia (1) 6/102 (2) 6/101 (3) 33/99<br>EP syndrome (1) 6/102 (2) 6/101 (3) 35/99

								>ULN<br>(1) 25/175<br>(2) 97/169<br>Mean dec in (1) and inc in (2)		cholesterol -no clin meaningful diff	EP rel (1) 42/175 (2) 108/172<br>EP syndrome (1) 16/175 (2) 61/172<br>Tremor (1) 12/175 (2) 17/169<br>Akathisia (1) 20/175 (2) 40/169
(1) 21/125<br>(2) 19/126	(1) 19/125<br>(2) 19/126	(1) 23/125<br>(2) 21/126	(1) 20/125<br>(2) 17/126		(1) 23/125<br>(2) 27/126	(1)13/125<br>(2) 14/126			abnormally high glucose<br>(1) 0/113<br>(2) 0/114	abnormally high cholesterol<br>(1) 1/118<br>(2) 0/115	Akathisia (1) 12/125 (2) 2/126<br>Tremor (1) 15/125 (2) 14/126
								prolactin measured but not reported	No case of clin relevant inc (<11.1 to ≥11.1 mmol/l)	cholesterol - no case of clin relevant inc (<5.17 mmol/l to ≥ 6.20 mmol/l)	Tremor (1) 7/51 (2) 4/48
									<200 to ≥200 mg/dl<br>(1)8/206<br>(2) 2/198	cholesterol <200 to ≥240 mg/dl<br>(1) 4/54<br>(2) 1/55	not reported
				(1) 14/225<br>(2) 2/136				(1) 12/104<br>(2) 2/64	<200 to ≥200 mg/dl<br>(1) 3/206<br>(2) 2/122	cholesterol <200 to ≥240 mg/dl<br>(1) 2/64<br>(2) 0/45	not reported
			(1) 6/77<br>(2) 9/83		(1) 7/77<br>(2) 6/83			(1) mean dec<br>(2) mean dec diff just sig	no sig diff betseen groups	no sig diff between groups	Akathisia (1) 5/77 (2) 1/83<br>Tremor (1) 7/77 (2) 1/83<br>Hypertonia (1) 3/77 (2) 1/83
							≥30 mm Hg dec in syst BP<br>(1) 5/346<br>(2) 6/82<br>(3) 5/352	no data	Mean changes sig greater in both olanzapine groups (4 to 6 mg/dl)<br><200 to ≥200 mg/dl:<br>(1) 4/289<br>(2) 1/65<br>(3) 1/298	Cholesterol - mean baseline levels high, with inc in both olanzapine groups (6 to 10 mg/dl)	
	(1) 1/10<br>(2) 0/10<br>(3) 0/10	(1) 0/10<br>(2) 1/10<br>(3) 1/10	(1) 0/10<br>(2) 0/10<br>(3) 1/10	(1) 2/10<br>(2) 2/10<br>(3) 1/10				no data	no data	no data	

(1) 20/180<br>(2) 21/179<br>(3) 8/180	(1) 41/180<br>(2) 30/179<br>(3) 15/180			no clinically relevant differences between groups in mean changes from baseline	no clinically relevant differences between groups in mean changes from baseline	no clinically relevant differences between groups in mean changes from baseline	EP-related (1) 16/180 (2) 12/179 (3) 4/180
	(1) 28/205<br>(2) 16/204		(1) 17/205<br>(2) 11/204	inc in mean from baseline with (1) but not (2)	no sig diff in mean change from baseline	Sig diff in mean change from baseline for cholesterol and triglycerides in favour of lamotrigine	Tremor (1) 22/205 (2) 3/204
(1) 17/168<br>(2) 14/171<br>(3) 5/167	(1) 27/168<br>(2) 24/171<br>(3) 9/167		(1) 19/168<br>(2) 16/171<br>(3) 13/167		No significant differences between groups in mean changes from baseline		EP symptoms (1) 17/168 (2) 21/171 (3) 11/167
