# Supplementary material for: Atypical antipsychotics in bipolar disorder: systematic review of randomised trials
Source: BMC Psychiatry. 2007 Aug 16;7:40. doi: 10.1186/1471-244X-7-40 (PMC2020469; doi:10.1186/1471-244X-7-40)
Supplement: Additional file 4 — Excluded papers. Papers excluded, with reason for exclusion. [file 1471-244X-7-40-S4.pdf]

Additional file 4: Excluded studies

Author	Publication	Reason for exclusion
Baker RW, et al.	The impact of response to previous mood stabilizer therapy on response to olanzapine versus placebo for acute mania. Bipolar Disord 2002;4:43-9.	Subgroup analysis of Tohen 2000
Namjoshi MA, et al.	Economic, clinical, and quality-of-life outcomes associated with olanzapine treatment in mania. Results from a randomized controlled trial. J Affect Disord 2002;69:109-18.	Same patients as Tohen 1999, with open-label extension
Shi L, et al.	Olanzapine versus haloperidol in the treatment of acute mania: clinical outcomes, health-related quality of life and work status. Int Clin Psychopharmacol 2002;17:227-37.	Same patients as Tohen 2003
Baker RW, et al.	Placebo-controlled trials do not find association of olanzapine with exacerbation of bipolar mania. J Affect Disord 2003;73:147-53.	Pooled secondary analysis
Baker RW, et al.	Acute dysphoric mania: treatment response to olanzapine versus placebo. J Clin Psychopharmacol 2003;23:132-7.	Pooled secondary analysis
Baldessarini RJ, et al.	Olanzapine versus placebo in acute mania: treatment responses in subgroups. J Clin Psychopharmacol 2003;23:370-6.	Pooled secondary analysis
Chengappa KN, et al.	Rates of response, euthymia and remission in two placebo-controlled olanzapine trials for bipolar mania. Bipolar Disord 2003;5:1-5.	Pooled secondary analysis
Hirschfeld RM, et al	The safety and early efficacy of oral-loaded divalproex versus standard-titration divalproex, lithium, olanzapine, and placebo in the treatment of acute mania associated with bipolar disorder. J Clin Psychiatry 2003;64:841-6.	Pooled secondary analysis
Sanger TM, et al.	Olanzapine in the acute treatment of bipolar I disorder with a history of rapid cycling. J Affect Disord 2003;73:155-61.	Subbgroup analysis
Yatham LN, et al.	Risperidone in acute and continuation treatment of mania. Int Clin Psychopharmacol 2003;18:227-35.	Patients incl in Yatham 2003
Baker RW, et al.	Efficacy of olanzapine combined with valproate or lithium in the treatment of dysphoric mania. Br J Psychiatry 2004;185:472-8.	Subgroup analysis
Namjoshi MA, et al.	Quality of life assessment in patients with bipolar disorder treated with olanzapine added to lithium or valproic acid. J Affect Disord 2004;81:223-9.	Same patients as Tohen 2003, not ITT
Shi L, et al.	Effects of olanzapine alone and olanzapine/fluoxetine combination on health-related quality of life in patients with bipolar depression: secondary analyses of a double-blind, placebo-controlled, randomized clinical trial. Clin Ther 2004;26:125-34.	Same patients as Tohen 2003
Vieta E, et al.	Comparison of rapid-cycling and non-rapid-cycling bipolar I manic patients during treatment with olanzapine: analysis of pooled data. J Clin Psychiatry 2004;65:1420-8.	Pooled secondary analysis
Amsterdam JD, et al.	Comparison of fluoxetine, olanzapine, and combined fluoxetine plus olanzapine initial therapy of bipolar type I and type II major depression--lack of manic induction. J Affect Disord 2005;87:121-30.	Fewer than 10 patients pre treatment group
Keck PE, et al.	Analyses of treatment-emergent mania with olanzapine/fluoxetine combination in the treatment of bipolar depression. J Clin Psychiatry 2005;66:611-6.	Same patients as Tohen 2003 (depression)
Strakowski SM, et al.	Quality of life during treatment with haloperidol or olanzapine in the year following a first psychotic episode. Schizophr Res 2005;78:161-9.	Patients with schizophrenia
Vieta E, et al.	Quetiapine monotherapy for mania associated with bipolar disorder: combined analysis of two international, double-blind, randomised, placebo-controlled studies. Curr Med Res Opin 2005;21:923-34.	Pooled secondary analysis
Gopal S, et al.	Symptomatic remission in patients with bipolar mania: results from a double-blind, placebo-controlled trial of risperidone monotherapy. J Clin Psychiatry 2005;66:1016-20.	Secondary analysis of Khanna, 2005
Nierenberg AA, et al.	Treatment-Resistant Bipolar Depression: A STEP-BD Equipoise Randomized Effectiveness Trial of Antidepressant Augmentation With Lamotrigine, Inositol, or Risperidone. Am J Psychiatry 2006;163:210-6.	Open-label treatment
